# The US President’s Malaria Initiative and under-5 child mortality in sub-Saharan Africa: A difference-in-differences analysis

**DOI:** 10.1371/journal.pmed.1002319

**Published:** 2017-06-13

**Authors:** Aleksandra Jakubowski, Sally C. Stearns, Margaret E. Kruk, Gustavo Angeles, Harsha Thirumurthy

**Affiliations:** 1 Department of Health Policy and Management, Gillings School of Global Public Health, University of North Carolina at Chapel Hill, Chapel Hill, North Carolina, United States of America; 2 Department of Global Health and Population, Harvard T.H. Chan School of Public Health, Harvard University, Boston, Massachusetts, United States of America; 3 Carolina Population Center, University of North Carolina at Chapel Hill, Chapel Hill, North Carolina, United States of America; 4 Department of Maternal and Child Health, University of North Carolina at Chapel Hill, Chapel Hill, North Carolina, United States of America; Mahidol-Oxford Tropical Medicine Research Unit, THAILAND

## Abstract

**Background:**

Despite substantial financial contributions by the United States President’s Malaria Initiative (PMI) since 2006, no studies have carefully assessed how this program may have affected important population-level health outcomes. We utilized multiple publicly available data sources to evaluate the association between introduction of PMI and child mortality rates in sub-Saharan Africa (SSA).

**Methods and findings:**

We used difference-in-differences analyses to compare trends in the primary outcome of under-5 mortality rates and secondary outcomes reflecting population coverage of malaria interventions in 19 PMI-recipient and 13 non-recipient countries between 1995 and 2014. The analyses controlled for presence and intensity of other large funding sources, individual and household characteristics, and country and year fixed effects.

PMI program implementation was associated with a significant reduction in the annual risk of under-5 child mortality (adjusted risk ratio [RR] 0.84, 95% CI 0.74–0.96). Each dollar of per-capita PMI expenditures in a country, a measure of PMI intensity, was also associated with a reduction in child mortality (RR 0.86, 95% CI 0.78–0.93). We estimated that the under-5 mortality rate in PMI countries was reduced from 28.9 to 24.3 per 1,000 person-years. Population coverage of insecticide-treated nets increased by 8.34 percentage points (95% CI 0.86–15.83) and coverage of indoor residual spraying increased by 6.63 percentage points (95% CI 0.79–12.47) after PMI implementation. Per-capita PMI spending was also associated with a modest increase in artemisinin-based combination therapy coverage (3.56 percentage point increase, 95% CI −0.07–7.19), though this association was only marginally significant (*p* = 0.054). Our results were robust to several sensitivity analyses. Because our study design leaves open the possibility of unmeasured confounding, we cannot definitively interpret these results as causal.

**Conclusions:**

PMI may have significantly contributed to reducing the burden of malaria in SSA and reducing the number of child deaths in the region. Introduction of PMI was associated with increased coverage of malaria prevention technologies, which are important mechanisms through which child mortality can be reduced. To our knowledge, this study is the first to assess the association between PMI and all-cause child mortality in SSA with the use of appropriate comparison groups and adjustments for regional trends in child mortality.

## Abstract 2

In a difference-in-difference analysis, Aleksandra Jakubowski and colleagues show that the introduction of the US President's Malaria Initiative in 19 African countries was associated with a significant reduction in the annual risk of under-5 child mortality.

## Author summary

**Why was this study done?:**

- Despite the considerable investment the US government has made in the President’s Malaria Initiative (PMI) since 2006, no studies to date have evaluated its association with population health outcomes.
- Previous evaluations have documented decreasing child mortality and increasing use of key malaria interventions in PMI-recipient countries. Our study sought to determine whether the trends in health outcomes in PMI-recipient countries differed significantly from the trends in these outcomes in PMI non-recipient countries in sub-Saharan Africa (SSA) over the past 2 decades.

**What did the researchers do and find?:**

- We used a study design that leveraged multiple publicly available data sources from countries throughout SSA, spanning the years before and after PMI introduction, in order to estimate association between the introduction of PMI and child mortality rates.
- Our dataset included 7,752,071 child-year observations from 2,112,951 individual children who lived in 32 sub-Saharan countries, including all 19 PMI countries.
- We found that after adjusting for baseline differences between countries, overall time trends, other funding sources, and individual characteristics, PMI was associated with 16% annual risk reduction in child mortality and increased population coverage of key malaria prevention and treatment technologies.
- We tested the robustness of our results with a series of sensitivity analyses.

**What do these findings mean?:**

- The study provides evidence that introduction of PMI was associated with significant reductions in child mortality in SSA, primarily through increased access to malaria prevention technologies.
- Evidence from this study can be used to inform policy decisions about future funding levels for malaria interventions.
- The interpretation of our study results rests on the assumption that there were no important unmeasured variables that differentially affected mortality rates in PMI and comparison countries during the study period.

## Introduction

At the turn of the millennium, malaria was the leading cause of child mortality in sub-Saharan Africa (SSA), claiming 694,000 child lives annually [1] and accounting for nearly a quarter of all under-5 deaths [2]. Reducing child mortality and lowering the burden of malaria were central components of the Millennium Development Goals and the key mission of the Roll Back Malaria Partnership [3, 4]. Since then, under-5 mortality has declined substantially in SSA, with all-cause child mortality rates declining from an estimated 158 deaths per 1,000 live births in 2000 to 82 deaths per 1,000 live births in 2015 in malaria-endemic countries, when malaria fell to the fourth leading cause of child deaths in SSA [1]. Understanding the role global policies and funding played in reducing malaria mortality, including changes in heath behaviors, can be valuable as countries and global donors work toward the Sustainable Development Goals, including the goal of eradicating malaria by 2030. While external funding remains a significant source of total health expenditures in low-income countries [5], health aid donations have stagnated since 2010 and future funding is surrounded by uncertainty [6]. The increasingly limited resources for healthcare delivery in developing countries demand strong evidence on the most effective and efficient ways to provide life-saving, evidence-based prevention and treatment interventions to vulnerable populations.

The US President’s Malaria Initiative (PMI), launched in 2005 by President George W. Bush and expanded by President Barack Obama, has been among the main sources of funding for malaria interventions in SSA [1, 6, 7]. With an annual budget of over $500 million since 2010 [8], PMI has primarily focused on provision of 4 recommended, evidence-based malaria interventions: insecticide-treated nets (ITNs) [9–11], rapid diagnostic tests and artemisinin-based combination therapy (ACTs) [10, 12], intermittent preventive treatment in pregnancy (IPTp) [11, 13–15], and indoor residual spraying (IRS) [11, 16]. Despite this sizable investment, the association between PMI, child mortality rates, and population-level coverage of key malaria interventions has not been previously examined.

While PMI program evaluations show that all-cause child mortality declined significantly in recipient countries [8, 17, 18], comparison groups are needed to determine whether these reductions were due to expansion in PMI funding or other interventions instead, including programs supported by the Global Fund for HIV/AIDS, Tuberculosis, and Malaria (Global Fund henceforth), the President’s Emergency Plan for AIDS Relief (PEPFAR), or domestic investments. Previous studies have documented higher access to ITNs, ACTs, and IRS coverage in PMI-recipient countries [19–22] but have not compared these trends to non-recipient countries. More rigorous analysis of the association between PMI and key outcomes is needed to assess whether PMI was successful at curbing child mortality through implementation of evidence-based malaria interventions. We used data from 32 sub-Saharan countries spanning nearly 20 years to determine the association between PMI and all-cause child mortality rates as well as malaria prevention and treatment behaviors.

## Methods

PMI began as a small program in 2006 with funding initially going to 3 countries and an annual budget of $30 million. Within 2 years, PMI scaled-up to 15 focus countries and had an annual budget of $300 million. By 2011, PMI expanded to 19 countries in SSA (Angola, Benin, Democratic Republic of Congo, Ethiopia, Ghana, Guinea, Kenya, Liberia, Madagascar, Malawi, Mali, Mozambique, Nigeria, Rwanda, Senegal, Tanzania, Uganda, Zambia, and Zimbabwe) and had an annual budget of $600 million. Approximately 40% of PMI’s budget has been allocated to procurement of commodities to prevent, diagnose, and treat malaria [8]. Since its inception, PMI has also focused on building capacity through training of healthcare workforce, providing technical support, and strengthening supply chain systems.

PMI country selection was based on several criteria, including high malaria burden, government capacity, potential for impact, willingness to partner with US government, national malaria control policies consistent with the World Health Organization standards, and other donor involvement in malaria control. Yet these criteria do not fully explain country selection. Some countries with relatively low malaria prevalence were selected (e.g., Ethiopia at 3.4% *Plasmodium falciparum* parasite rate in 2- to 10-year-olds [P*f*Pr_2-10_] in 2005 and Zimbabwe at 2.5% P*f*Pr_2-10_ in 2005) whereas others with high malaria burden were not selected (e.g., Cameroon at 47.0% P*f*Pr_2-10_ in 2005 and Burkina Faso at 55.4% P*f*Pr_2-10_ in 2005). Cameroon and Burkina Faso also score higher on World Bank’s Worldwide Governance Indicators than do Ethiopia and Zimbabwe. Thus, while selection process of PMI-recipient countries was not random, it does not appear to have been systematically associated with malaria burden, governance, or health system performance. The analyses carried out in this study, described below, include comparisons of recipient and non-recipient countries as well as tests for differential impacts based on initial country characteristics.

### Measures

#### Child mortality data

Child mortality data were obtained from 77 Demographic and Health Surveys (DHS), 14 Malaria Indicator Surveys (MIS), and 5 AIDS Indicator Surveys (AIS) in 32 countries in SSA. DHS, MIS, and AIS are nationally representative, cross-sectional surveys that include common questions about birth dates and survival status of all births to women of reproductive age (15–49 years) [23]. From these data, we constructed a longitudinal cohort with annual observations for each live birth between 1995 and 2014. We used information about the year of child’s birth, whether each child was alive at the time of the survey, and how old a child was if s/he died to define the primary outcome, which was a binary indicator of mortality in each year. We also extracted data about the child’s age and gender, mother’s age, mother’s education, mother’s parity, household wealth, whether the head of household was female, and whether the household was in rural area. We excluded from the analysis malaria non-endemic countries (Lesotho), small island countries (Comoros, Sao Tome, and Principe) and South Africa, because only one DHS from 1998 was available there. Additional details about data structure are described in S1 Appendix.

#### Malaria interventions coverage data

Secondary outcomes were defined based on country-level annual data about population coverage of key malaria prevention and treatment interventions, which we obtained from the Malaria Atlas Project (MAP) [24]. MAP estimates of ITN, ACT, and IRS coverage are based on household-level data from DHS, MIS, Multiple Indicator Cluster Surveys, AIS, Malaria and Anemia Prevalence Survey, and the World Health Organization, combined with national malaria control program data from 43 countries between 2000 and 2015 [10]. The ITN estimate represents the proportion of people who slept under an insecticide-treated bednet on any given night each year; the ACT estimate represents the proportion of fever cases in under-5-year-olds receiving ACTs; and the IRS estimate represents the proportion of the population protected by indoor spraying of insecticides. We did not find reliable data on rapid diagnostic tests and IPTp.

### Exposure to PMI and other health aid

We measured receipt of donor funding by individual countries with 2 measures: a binary indicator of whether a program provided funding to a given country in a given year and a continuous measure of program intensity using per-capita disbursements in a given country and year. The binary indicators of program activity were extracted from reports to US Congress. The per-capita aid measures address the possibility that program activities vary in scope between countries and within countries over time. These country-year data were obtained from publicly available Development Assistance for Health 1990–2014 dataset, compiled by the Institute for Health Metrics and Evaluation (IHME) [25]. We excluded 2013–2014 disbursements from the analysis, as these data were not complete due to reporting lags. The IHME dataset distinguished between the funding source (country of origin), the distribution channel (bilateral versus multilateral versus private foundations), health focus area, and the recipient country. Using these characteristics, we divided total development assistance for health into 6 categories (details in the S1 Appendix): PMI (US bilateral aid for malaria); Global Fund malaria aid; other malaria aid; PEPFAR (US bilateral aid for HIV/AIDS); Global Fund HIV/tuberculosis aid; and all other health disbursements. These 6 categories summed to 100% of development assistance for health provided to SSA that were captured in IHME dataset. All health disbursements in this study are reported in 2014 US dollars.

### Statistical analyses

We performed difference-in-differences analysis by estimating regression models that compared trends in outcomes in PMI recipient counties to trends in comparison countries while adjusting for country fixed effects, which controlled for underlying differences between countries, and year fixed effects, which controlled for secular trends in outcomes. We evaluated the primary outcome of annual child mortality risk using modified Poisson regression models [26] with robust standard errors clustered at the country level to relax the assumption of independently and identically distributed error terms [27, 28]. The proportion of population covered by ITN, IRS, and ACT outcomes were evaluated using ordinary least squares regression models, with robust standard errors. Statistical significance threshold was set at alpha = 0.05 using two-tailed tests. We used child-year data from the DHS, MIS, and AIS to estimate how binary indicators of program activity were associated with annual risk of mortality among children aged <5 years. The first model included a binary indicator of PMI funding in a given country and year (Model 1 in S1 Appendix). The second model added binary indicators for whether countries received funding from other large-scale donors in each year, namely the Global Fund and PEPFAR (Model 2). The third model also added individual and household characteristics (Model 3); observations with missing data were dropped from analysis. We then re-fitted these 3 models using our continuous measures of program intensity, or per-capita aid disbursements, instead of the binary funding indicators (Models 4–6). A similar analytical strategy was undertaken in a previous study that sought to estimate the effect of PEPFAR on adult mortality in SSA [29].

Country-level MAP data were then used to evaluate the association between binary indicators of PMI, PEPFAR and Global Fund, and population-coverage of ITNs, ACTs, and IRS, while controlling for total population size (Model 7). Next, we explored the associations between per-capita measures of program intensity and malaria intervention coverage, again controlling for population size (Model 8). Finally, we tested whether the associations between PMI and health-related outcomes varied over time by including a series of binary indicators for each year in which PMI, Global Fund, and PEPFAR funds were available in a country (Models 9 and 10). These models were used to test the mechanism though which introduction of PMI funding might have shifted trends in malaria incidence and under-5 mortality rates.

The difference-in-differences design is a quasi-experimental method that relies on the assumption that, in the absence of any intervention, countries receiving PMI funding would have identical trends in outcomes as non-recipient countries. We tested this “parallel trends” assumption by using data from pre-PMI years and estimating models that included an interaction term between an indicator of PMI country and a linear time trend (Model 11). In order to assess whether countries were selectively chosen for PMI, we also compared the average baseline performance on the available measures of governance and health systems between PMI recipient and comparison countries [30].

We performed several sensitivity analyses to verify our results. First, we excluded deaths that occurred in the first month of the child’s life to confirm that results were not driven by reductions in neonatal mortality. Reductions in neonatal mortality could be attributed to better prenatal and delivery care rather than malaria interventions. Second, we tested whether the association between PMI and child mortality was stronger in rural areas where malaria burden is generally higher. Third, we separately excluded each individual country from the analysis to ensure that results were not driven by patterns in any single country. Fourth, we assessed whether results were robust to excluding the Democratic Republic of Congo and Nigeria because PMI programs were implemented at subnational levels in these 2 countries and under-5 mortality rates due to malaria were especially high there [31]. Fifth, we assessed whether the results were robust to the type of model chosen by also estimating logit, probit, and Cox regression models. Next, we confirmed that the parallel trends assumption held when we interacted a nonlinear time trend with PMI program indicators using data from pre-PMI years. Finally, we confirmed that we were able to detect the association between PMI and malaria intervention coverage using alternative data sources.

## Results

The child mortality sample from DHS, MIS, and AIS included 7,752,071 child-year observations, of which 5,837,998 (75%) were from 19 PMI-recipient countries and the remaining 1,914,073 (25%) were from 13 comparison countries (Table 1). Approximately 9.44% of the 2,112,951 children in our sample died before reaching age 5. In PMI countries, we observed 148,551 child deaths during the study period (under-5 mortality rate 25.4 per 1,000 person-years) while in comparison countries we observed 50,930 child deaths (under-5 mortality rate of 26.6 per 1,000 person-years). Detailed information about program start years, survey years, and sample size by study country are provided in Table A of S1 Appendix. Characteristics of children and mothers in the sample did not significantly differ between PMI recipient and non-recipient countries, with 2 exceptions: fewer women in PMI countries disclosed having no education and a larger share of children in PMI countries lived in rural areas (Table B in S1 Appendix). The data on coverage of malaria-related interventions included 512 country-year observations, of which 304 (59%) were from PMI countries and 208 (41%) from comparison countries.

**Table 1:** Description of study sample.

Description of study sample	PMI countries	Comparison countries	Full sample
Description of under-5 mortality study sample (DHS data)	Frequency	Frequency	Frequency
Number of child-year observations	5,837,998	1,914,073	7,752,071
Child-year observations after PMI implementation	1,266,884	0	1,266,884
Child-year observations after Global Fund	3,014,262	929,326	3,943,588
Child-year observations after PEPFAR	1,650,871	137,842	1,788,713
Number of individual children in sample	1,586,824	526,127	2,112,951
Number of children under 5 years who died	148,551	50,930	199,481
Child mortality rate (per 1,000 person-years)	25.4	26.6	25.7
**Description of malaria interventions coverage sample (MAP data)**			
Number of country-year observations	304	208	512
Country-year observations after PMI implementation	150	0	150
Country-year observations after Global Fund	243	160	403
Country-year observations after PEPFAR	143	43	186
Number of individual countries in sample	19	13	32

**Abbreviations**: DHS, Demographic and Health Surveys; MAP, Malaria Atlas Project; PEPFAR, President’s Emergency Plan for AIDS Relief; PMI, President’s Malaria Initiative.

**Notes:** DHS data from 1995-2014, MAP data from 2000-2015. PMI recipient countries (year of implementation): Angola (2006), Benin (2008), Congo DRC (2011), Ethiopia (2008), Ghana (2008), Guinea (2011), Kenya (2008), Liberia (2008), Madagascar (2008), Malawi (2007), Mali (2008), Mozambique (2007), Nigeria (2011), Rwanda (2007), Senegal (2007), Tanzania (2006), Uganda (2006), Zambia (2008), Zimbabwe (2011). Comparison countries: Burkina Faso, Burundi, Cameroon, Chad, Congo, Cote d’Ivoire, Gabon, Namibia, Niger, Sierra Leone, Swaziland, The Gambia, and Togo.

We found no major differences between PMI and comparison countries in baseline malaria burden, child mortality rates, and indicators of health system performance or governance (Table 2). While there was considerable variation across countries in baseline malaria burden, the average rate of *P*. *falciparum* in 2- to 10-year-olds in 2005 was approximately 30% in both study groups. The World Bank’s estimates of under-5 mortality rates [30] in 2005 were 121 per 1,000 live births in PMI countries and 128 per 1,000 live births in comparison countries (Table 2 and Table C in S1 Appendix). PMI countries had lower baseline health expenditures and fewer nurses and midwives than comparison countries, but these differences were not statistically significant. Both study groups had negative (i.e., unfavorable) scores on government effectiveness, political stability, and corruption measures, and the differences between groups in these scores were not statistically significant. PMI countries had larger (*p* = 0.03) and less wealthy (*p* = 0.052) populations at baseline. Country-level baseline under-5 mortality rates, PfPR_2-10_ transmission rates, and coverage of ITNs, ACTs, and IRS are listed in Table C in S1 Appendix. PMI recipient countries had higher ITN coverage at baseline, but ACT and IRS coverage was approximately the same in 2005 between the study groups. Our study design adjusts for baseline differences between countries.

**Table 2:** Description of study countries.

Description of countries in sample (MAP, WDI data)	PMI countries	Comparison countries	*p* value
Average *Plasmodium falciparum* rate in 2- to 10-year-olds 2005, %	30.0%	30.2%	0.980
Under-5 mortality rate in 2005 (per 1,000 live births), mean	121	128	0.541
Population in 2005 (in thousands), mean	28,713	7,790	0.030
Adult literacy rate in 2005, %	54	52	0.807
GNI per capita in 2005 (PPP), mean	$1,466	$4,015	0.052
Health expenditures in 2005 (PPP), mean	89	160	0.070
Physician density in 2005 (per 1,000 people), mean	0.10	0.11	0.680
Nurses and midwives density in 2005 (per 1,000 people), mean	0.66	1.45	0.108
Government effectiveness index in 2005, mean^a^	−0.79	−0.99	0.179
Political stability index in 2005, mean^b^	−0.71	−0.63	0.777
Corruption index in 2005, mean^c^	−0.79	−0.77	0.910

**Abbreviations:** %, Percen; GNI, Gross National Income; MAP, Malaria Atlas Project; PMI, President’s Malaria Initiative; PPP, purchasing power parity (in 2011 constant dollars); WDI, World Development Indicators.

**Data Sources:** Average *Plasmodium falciparum* rate obtained from the MAP. Under-5 mortality rate, population, adult Literacy, GNI per capita, health expenditures, physician and nurse density data obtained from the World Development Indicators, February 2017 version. Government effectiveness, Political stability and Corruption indexes obtained from The Worldwide Governance Indicators, 2015 Update.

^a^Government effectiveness reflects perceptions of the quality of public services, the quality of the civil service and the degree of its independence from political pressures, the quality of policy formulation and implementation, and the credibility of the government’s commitment to such policies.

^b^Political Stability measures perceptions of the likelihood of political instability and/or politically motivated violence, including terrorism.

^c^Corruption index reflects perceptions of the extent to which public power is exercised for private gain, including both petty and grand forms of corruption, as well as “capture” of the state by elites and private interests. Estimates of governance, corruption, and political stability range from approximately −2.5 (weak) to 2.5 (strong) performance.

The test of parallel trends showed that, after controlling for baseline country characteristics, secular time trends, and individual characteristics, child mortality rates in PMI recipient and non-recipient countries were identical *before* the start of the PMI program (risk ratio [RR] 1.00, 95% CI 0.98–1.01; Table 3). Mortality rates were declining in all of the study countries during this time period (RR 0.96, 95% CI 0.95–0.97), but there was no evidence of trends being different between PMI and comparison countries.

**Table 3:** Test of parallel trends assumption: Risk of child mortality prior to PMI program implementation.

	Annual risk of child mortality prior to PMI
Outcome	RR [95% CI]
PMI-recipient country	1.04 [0.96–1.13]
Linear time trend	0.96*** [0.95–0.97]
PMI-recipient country * time interaction	1.00 [0.98–1.01]
*Child’s characteristics*	
Female	0.89*** [0.87, 0.90]
Age (<1 year)	*Ref*.
Age (<2 years)	1.64*** [1.51–1.79]
Age (<3 years)	0.59*** [0.52–0.68]
Age (<4 years)	0.41*** [0.37–0.45]
Age (<5 years)	0.25*** [0.22–0.27]
*Mother’s characteristics*	
No education	*Ref*.
Primary education	0.89*** [0.86–0.93]
Secondary education	0.77*** [0.74–0.80]
Higher education	0.68*** [0.63–0.74]
Age	0.94*** [0.93–0.94]
Parity	1.18*** [1.17–1.19]
*Household characteristics*	
Rural residence	1.09*** [1.05–1.14]
Lowest wealth quintile	*Ref*.
Second wealth quintile	0.99 [0.94–1.04]
Middle wealth quintile	0.95 [0.91–1.00]
Fourth wealth quintile	0.88*** [0.82–0.95]
Highest wealth quintile	0.71*** [0.67–0.75]
Female household head	1.01 [0.99–1.03]
No. observations (children-years)	6,174,926

**Data Source:** Demographic and Health Surveys

**Abbreviations:** PMI, President’s Malaria Initiative; RR, adjusted risk ratio

**Notes:** PMI-recipient country variable indicates whether a country eventually received PMI funds. Linear time trend measures secular mortality trends. The coefficient of interest is the interaction of PMI country indicator and linear time trend, which measures whether the mortality trend in countries that eventually received PMI differed from mortality trend in comparison countries, adjusted for individual-level covariates. Model also included country fixed effects. Standard errors were clustered at the country level. Sample excludes observations from PMI-recipient countries after the program was implemented.

*** *p* < 0.001,

** *p* < 0.01,

* *p* < 0.05.

**Fig 1:**
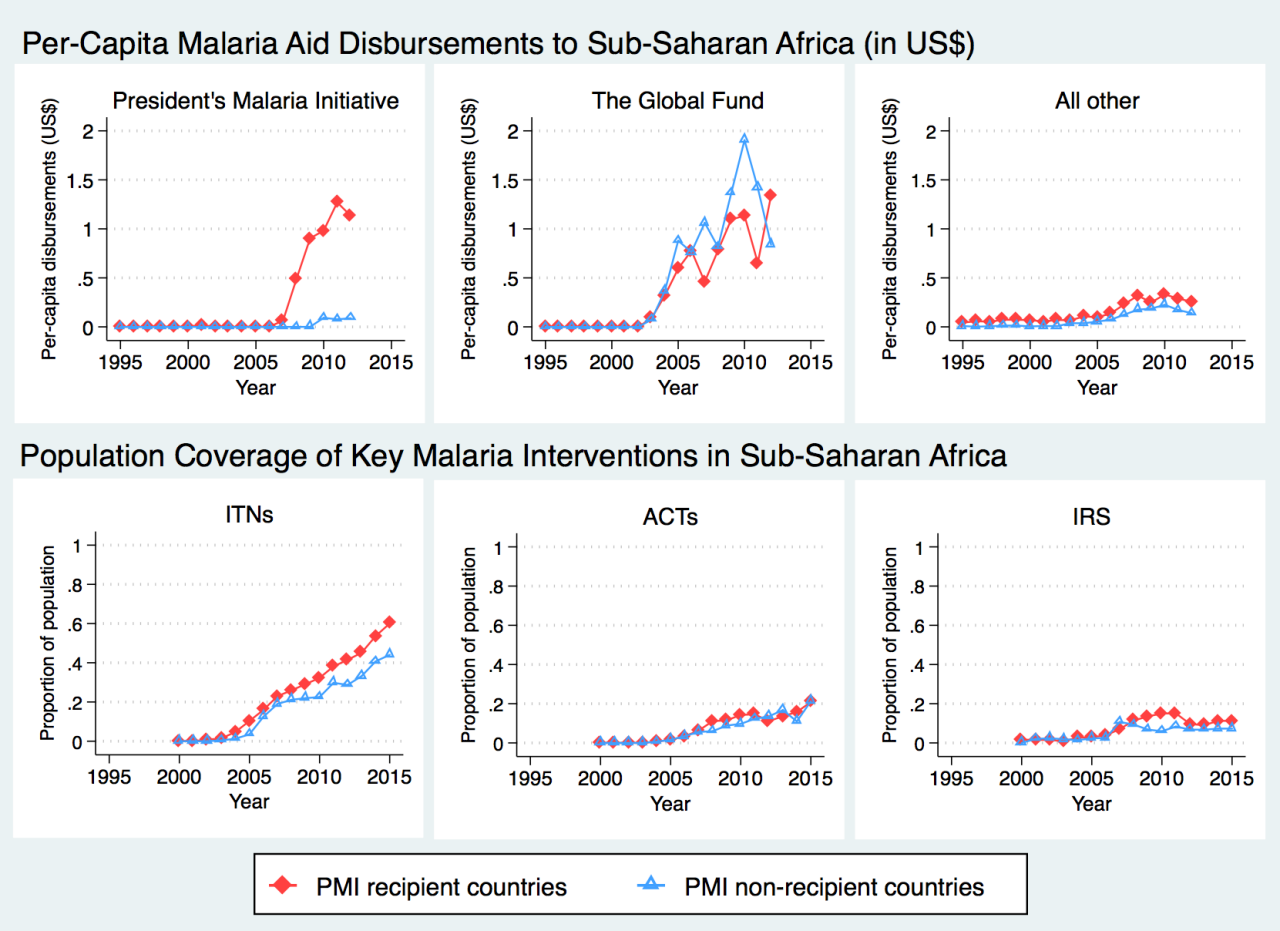
Time trends in development assistance for malaria and coverage of malaria interventions in sub-Saharan Africa. Total assistance for malaria, in 2014 US dollars, was divided into 3 categories: (1) US bilateral aid for malaria, a proxy for PMI disbursements; (2) Global Fund, limited here to malaria disbursements; (3) All other malaria aid includes total malaria aid minus US bilateral aid minus GF malaria aid. **Data Sources:** Development Assistance for Health (DAH) data from 1995–2012 were obtained from Institute for Health Metrics and Evaluation. Population coverage of ITNs, ACTs, and IRS from 2000–2015 were obtained from the Malaria Atlas Project. **Abbreviations:** ACTs, estimated proportion of cases of fever in under-5 year olds that were treated with artemisinin combination therapy; IRS, estimated proportion of the population protected by indoor residual spraying of insecticides; ITNs, estimated proportion of people who slept under an insecticide-treated bednet on any given night; PMI, President’s Malaria Initiative.

PMI countries received an average of US$0.98 per-capita from US bilateral aid for malaria annually (Fig 1). The Global Fund provided about twice as much aid to the sub-Saharan region as PMI, with about half of the disbursements sent to PMI-recipient countries (average of US$0.89 per capita since 2006) and the other half to non-recipient countries (average of US$1.01 per capita since 2006). PMI countries received slightly more malaria funding from all other sources than non-PMI countries (US$0.26 USD versus US$0.11, respectively, since 2006), though all other malaria aid summed to less than $0.50 per-capita at its highest level. The upward trend in ITN coverage was apparent during the study period, with larger increases observed in PMI countries. ACT coverage remained fairly low in all countries and did not differ between PMI recipient versus non-recipient countries. While average IRS coverage increased in PMI countries soon after the program was introduced, this trend was not sustained, and by 2012 average coverage in the 2 study groups was again overlapping.

The average annual risk of mortality among children aged <5 years was 15% lower after the introduction of PMI (Table 4, Panel A; risk ratio [RR], 0.85, 95% confidence interval, CI, 0.74–0.96). Result was slightly more pronounced, at 16% reduction in mortality, after adjusting for the presence of other funding sources in a country (RR 0.84, 95% CI 0.74–0.95) and in the fully adjusted model that included individual characteristics (RR 0.84, 95% CI 0.74–0.96). The main study finding of a 16% reduction in the annual risk of child mortality after adjustment for covariates amounts to a change in the mortality rate from 28.9 per 1,000 person-years in PMI countries before program implementation to 24.3 per 1,000 person-years after implementation. Per-capita measure of PMI intensity showed that each additional dollar disbursed through PMI was associated with a reduction in the annual risk of under-5 mortality (Table 4, Panel B; RR 0.84, 95% CI 0.77–0.90). After adjusting for other funding sources and individual-level covariates, PMI was associated with 14% annual reduction in under-5 mortality (RR 0.86, 95% CI 0.79–0.93). Per-capita Global Fund disbursements were marginally associated with lower risk of under-5 all-cause mortality (RR 0.96, 95% CI 0.93–1.00) in the fully adjusted models. Mortality risk was lower for females, children with more educated mothers, and children living in wealthier households and in urban areas (Tables D and E in S1 Appendix); children between first and second birthdays faced the highest risk of mortality.

**Table 4:** Association between PMI and annual risk of mortality among children <5 years in sub-Saharan Africa.

**Panel A: (binary measures of donors)**	**Annual risk of child mortality and binary program indicators**		
	(1)	(2)	(3)
	RR [95% CI]	RR [95% CI]	RR [95% CI]
Implemented program			
Post PMI implementation	0.85** **[0.74–0.96]**	0.84** **[0.74–0.95]**	0.84** **[0.74–0.96]**
Post Global Fund implementation		**0.95 [0.87–1.04]**	**0.93 [0.85–1.02]**
Post PEPFAR implementation		**1.06 [0.96–1.17]**	**1.05 [0.95–1.17]**
No. observations (children-years)	7,752,071	7,752,071	7,404,578
Individual covariates	No	No	Yes
**Panel B: (per-capita measures of donors)**	**Annual risk of child mortality and per-capita disbursements for health**		
	(4)	(5)	(6)
	RR [95% CI]	RR [95% CI]	RR [95% CI]
Per-capita aid disbursements (US$)			
PMI (US bilateral aid for malaria)	0.84*** **[0.77–0.90]**	0.85*** **[0.78–0.92]**	0.86*** **[0.79–0.93]**
Global Fund (malaria only)		0.96* **[0.93–1.00]**	**0.96 [0.93–1.00]**
Other aid for malaria		**1.04 [0.89–1.21]**	**1.04 [0.87–1.24]**
Global Fund (HIV/AIDS and TB)		**1.00 [0.96–1.03]**	**1.00 [0.96–1.03]**
PEPFAR (US bilateral aid for HIV/AIDS)		**1.01 [0.99–1.02]**	**1.01 [0.99–1.02]**
All other disbursements for health		**0.99 [0.98–1.01]**	**1.00 [0.98–1.01]**
No. observations (children-years)	7,140,735	7,140,735	6,829,406
Individual covariates	No	No	Yes

**Data sources:** Demographic Health Surveys from 1995 to 2014; Development Assistance for Health Database from 1995 to 2012, and World Development Indicators.

**Abbreviations**: PEPFAR, President’s Emergency Plan for AIDS Relief; PMI, President’s Malaria Initiative; RR, adjusted risk ratio; TB, tuberculosis.

**Notes:** Models (1) to (6) are briefly described in the Methods section and are described in detail in S1 Appendix. All models included country and year fixed effects and were calculated using robust standard errors. Models (4)–(6) are limited to 1995–2012 because estimates for Development Assistance for Health Database were not available for later years. Models (3) and (6) included individual-level covariates: child’s age and gender, mother’s level of education, age and parity, rural/urban residence, household wealth and whether the head of household is female. The full list of individual-level estimates and confidence intervals is displayed in Tables D and E in S1 Appendix.

*** *p* < 0.001,

** *p* < 0.01,

* *p* < 0.05.

PMI was also associated with 8.34 percentage point increase in ITN coverage (95% CI 0.86–15.83) and 6.63 percentage point increase of IRS coverage (95% CI 0.79–12.47), as shown in Panel A of Table 5. When we examined PMI on the basis of program intensity (Table 5, Panel B), each additional per-capita dollar disbursed through PMI was associated with 4.29 percentage point annual increase in ITN coverage (95% CI 0.54–8.03) and 3.56% increase in ACTs (−0.07–7.19), though this association was only marginally significant (*p* = 0.054). Despite the relatively small amount of malaria funds disbursed through channels other than PMI and Global Fund, we found that ITN coverage increased by 9.69 percentage points (95% CI 3.41–15.97) in countries that received funding through these channels. When the study sample was expanded beyond the 32 study countries that had DHS/MIS/AIS child mortality data (i.e., to other countries in SSA that also had MAP data), we found that PMI was associated with even larger increases in ITN, ACT, and IRS coverage (Table F in S1 Appendix).

**Table 5:** Population coverage of insecticide treated nets (ITNs), artemisinin-based combination therapy (ACTs), and indoor residual spraying (IRS) in 19 PMI-recipient countries compared to 13 non-recipient countries in sub-Saharan Africa.

**Panel A (binary measures of donors)**	**Models of population coverage of malaria interventions and program implementation**		
	**ITNs**	**ACTs**	**IRS**
	(7)	(7)	(7)
	Coef. [95% CI]	Coef. [95% CI]	Coef. [95% CI]
*Implemented program*			
Post PMI	8.34* [0.86–15.83]	2.98 [−3.18–9.14]	6.63* [0.79–12.47]
Post Global Fund	−5.91 [−13.33–1.51]	0.85 [−4.75–6.45]	1.79 [−2.97–6.55]
Post PEPFAR	−3.23 [−11.27–4.82]	1.30 [−3.02–5.62]	−1.06 [−4.88–2.77]
No. observations (country-years)	512	512	512
Average coverage in PMI countries before intervention	7.0%	1.1%	3.0%
**Panel B (per-capita measures of donors)**	**Models of population coverage of malaria interventions and per-capita disbursements for health**		
	**ITNs**	**ACTs**	**IRS**
	(8)	(8)	(8)
	Coef. [95% CI]	Coef. [95% CI]	Coef. [95% CI]
*Per capita aid disbursement (US$)*			
US bilateral aid for malaria	4.29* [0.54–8.03]	3.56 [−0.07–7.19]	1.98 [−1.32–5.27]
Other aid for malaria	9.69** [3.41–15.97]	1.70 [−4.40–7.80]	1.90 [−8.15–11.95]
Global Fund (malaria only)	1.51 [−0.02–3.05]	0.27 [−0.81–1.35]	0.11 [−1.33–1.54]
Global Fund (HIV/AIDS and TB)	−0.22 [−0.77–0.34]	−0.12 [−0.38–0.15]	0.61* [0.15–1.08]
US bilateral aid for HIV/AIDS	−0.39* [−0.73–−0.04]	−0.03 [−0.26–0.20]	−0.15 [−0.75–0.45]
All other disbursements for health	−0.39 [−1.18–0.39]	−0.15 [−0.74–0.44]	0.16 [−0.48–0.79]
No. observations (country-years)	416	416	416
Average coverage in PMI countries before intervention	7.0%	1.1%	3.0%

**Data sources:** Malaria Atlas Project (MAP), Development Assistance for Health Database (DAH), and World Development Indicators (WDI).

**Abbreviations:** ACTs, estimated proportion of cases of fever in under-5 year olds that were treated with artemisinin combination therapy; Coef., coefficient; IRS, estimated proportion of the population protected by indoor residual spraying of insecticides; ITNs, estimated proportion of people who slept under an insecticide-treated bednet on any given night; PEPFAR, President’s Emergency Plan for AIDS Relief; PMI, President’s Malaria Initiative; TB, tuberculosis.

**Notes**: Coefficients can be interpreted as percentage changes. All models also included country and year fixed effects and population size. Robust standard errors were used to calculate confidence intervals and *p* values. Models (7) and (8) are briefly described in the Methods section and are described in detail in S1 Appendix.

*** p<0.001,

** p<0.01,

* p<0.05.

We used estimates from prior studies to calculate the predicted reductions in all-cause child mortality based on the PMI-associated increases in ITN, ACT, and IRS coverage reported in Table 5. Our calculations incorporated evidence from Kenya, where Fegan et al. (2007) estimated that increasing ITN coverage from 7% to 67% was associated with 44% reduction in all-cause child mortality [32]. Given the limited evidence about the impact of IRS on child mortality [16], we followed the example of Eisele et al. (2010) and assumed that IRS coverage had approximately equal protective effect to ITNs [11]. Finally, we used evidence from Zanzibar, where Bhattarai et al. (2007) found that reaching high coverage of ACTs (we used the conservative assumption that this implied full coverage) was associated 52% reduction in child mortality [33]. Applying these effect sizes to the estimates in Table 5, we determined that the increased coverage of these 3 prevention and treatment modalities could account for a 12.5% reduction in all-cause child mortality (additional details are in S1 Appendix).

**Fig 2:**
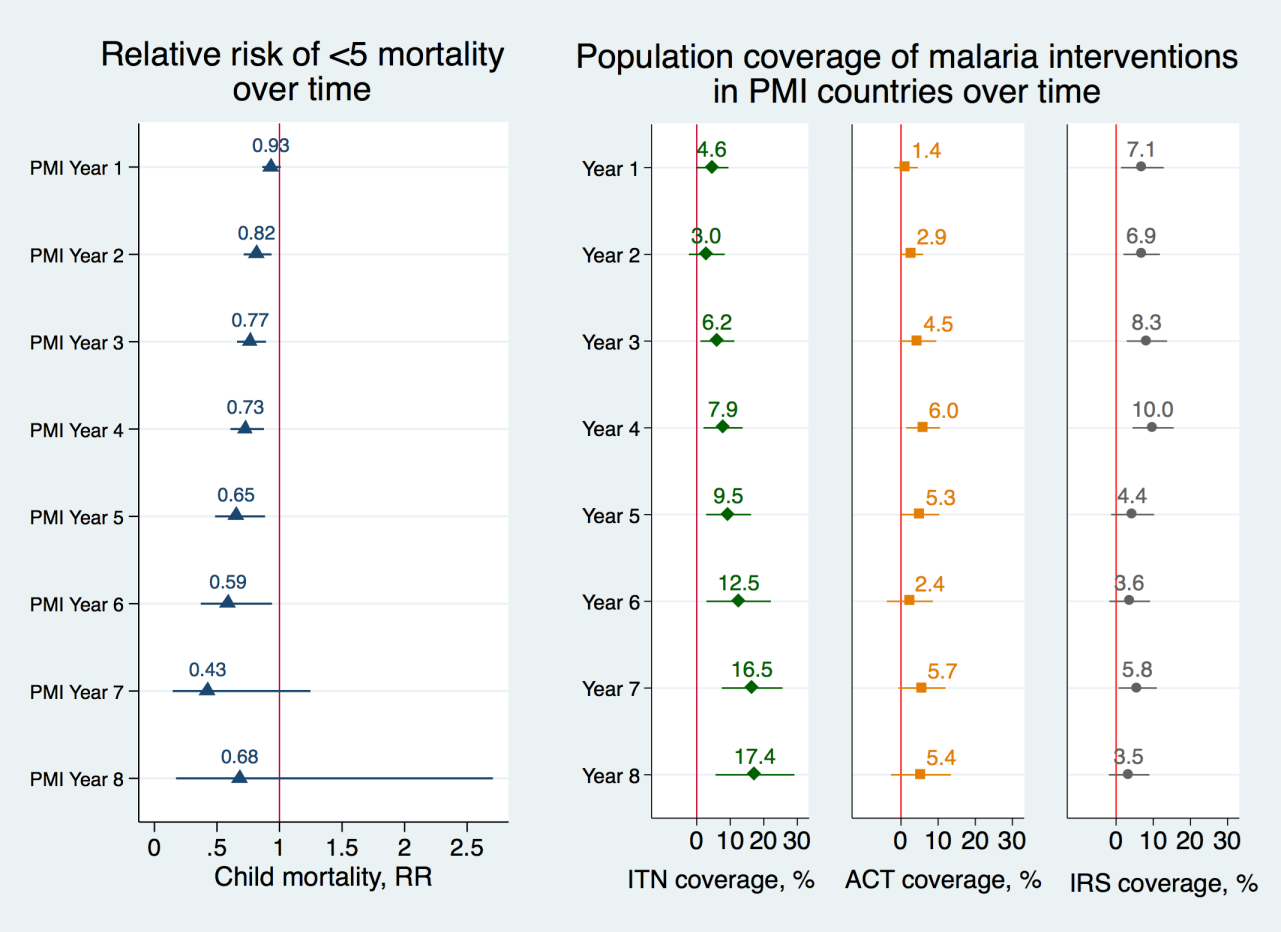
Adjusted risk ratios of child mortality and adjusted percentage changes in population coverage of malaria interventions as a function of year of PMI program implementation. Risk ratios of child mortality were estimated using modified Poisson regression model controlling for a set of indicators of the year of PEPFAR implementation, a set of indicators of the year of Global Fund implementation, individual-level covariates, country and year fixed effects (Model 9 in S1 Appendix). Standard errors were clustered at the country level. Error bars represent 95% confidence intervals. Changes in ITN, ACT, and IRS coverage were obtained using OLS regression models, controlling for a set of indicators of the year of PEPFAR implementation, a set of indicators of the year of Global Fund implementation, individual-level covariates, country and year fixed effects (Model 10) and robust standard errors. PMI year 9 was omitted from the figure because too few observations were available for the calculations (full list of coefficients and confidence intervals is listed in Table G in S1 Appendix). **Data Sources:** Demographic and Health Surveys from 1995–2014; Malaria Atlas Project from 2000–2014. **Abbreviations:** ACTs, estimated proportion of cases of fever in under-5 year olds that were treated with Artemisinin Combination Therapy; IRS, estimated proportion of the population protected; ITNs, estimated proportion of people who slept under an insecticide-treated bednet on any given night; PMI, President’s Malaria Initiative; RR, adjusted risk ratio.

Fig 2 displays the association between PMI and child mortality over time and the association between PMI and coverage of malaria interventions over time. We found that as the number of years of PMI implementation rose, there were larger associated reductions in mortality rates in PMI countries and larger associated increases in coverage of ITNs. In the first year of PMI implementation, the association between the program and child mortality was small and not statistically significant (RR 0.93, 95% CI 0.86–1.01) but in subsequent years the association increased considerably (RR 0.73, 95% CI 0.61–0.88 in year 4 and RR 0.65, 95% CI 0.48–0.88 in year 5), full set of results in Table G in S1 Appendix. The coefficients in year 7 and 8 have wide confidence intervals because they are based on a much lower number of observations due to fewer countries having been enrolled at the start of PMI in 2006 and fewer DHS surveys available from the later years. Similarly, the association between PMI and ITN coverage began as a small increase, but by the fifth year of PMI, the association increased to 9.5 percentage points (95% CI 2.8–16.2) and reached a peak of approximately 17 percentage point increase in years 7 and 8 (95% CI 7.5–25.6 and 5.6–29.2, respectively). IRS coverage was increasing in PMI-recipient countries up to the fourth year of program implementation, at which point it dropped off considerably. ACT coverage increased at very moderate levels in PMI countries throughout this program’s history.

We tested the robustness of study results with a series of sensitivity analyses. When neonatal deaths (i.e., those that took place within the first month of a child’s life) were excluded from the analysis, the association between PMI and all-cause child mortality rate was magnified from RR 0.84 to a RR of 0.79 (95% CI 0.69–0.90) and each additional per-capita dollar spent through PMI was associated with 0.83 lower risk of mortality (95% CI 0.75–0.92), results listed in Table H in S1 Appendix. Reductions in child mortality were especially evident in rural areas, where malaria burden is typically the highest and where access to malaria interventions has the higher potential for impact. The annual risk reduction of child mortality was 0.83 (95% CI 0.73–0.95) in rural areas compared to 0.87 (95% CI 0.76–1.00) in urban areas (results listed in Table I in S1 Appendix). Each additional per-capita dollar disbursed through PMI was associated with 0.85 lower risk reduction of mortality in rural areas (95% CI 0.78–0.93) compared to 0.88 (95% CI 0.82–0.93) in urban areas. Results were also robust to excluding individual countries, to excluding Democratic Republic of Congo and Nigeria from the model (Table J in S1 Appendix), and to different model specifications (Table K in S1 Appendix). We confirmed that the parallel trends assumption held when we interacted nonlinear time trend with PMI indicators (Table L in S1 Appendix). Finally, we confirmed our study finding that PMI was associated with increased utilization of ITNs using alternative data sources (Table M in S1 Appendix).

## Discussion

This study evaluated the association between PMI and population health using methods that controlled for various confounding factors. PMI was associated with large and statistically significant reductions in all-cause mortality rates among children under 5 years of age. These findings persisted in models that controlled for the presence and size of funding from other important programs, time-invariant country characteristics, common time trends, and various individual and household characteristics. Among other funders, disbursements through the Global Fund were also modestly associated with all-cause child mortality rates. The results suggest that PMI’s investment in key malaria interventions was associated with significant increases in population coverage of malaria prevention and treatment technologies, which ultimately may have contributed to the significant reductions in under-5 mortality in SSA.

The main findings indicate that PMI was associated with a 16% decline in annual risk of all-cause under-5 mortality. Furthermore, the association between PMI and child mortality was more pronounced over time. These reductions were above and beyond the declines in child mortality that were observed in PMI and non-PMI countries prior to the introduction of PMI, as well as trends in mortality that were observed in non-PMI countries in the years after introduction of PMI. The 16% relative risk reduction translates to a decline in under-5 mortality rate from 28.9 to 24.3 deaths per 1,000 person-years after PMI implementation. Because the primary outcome in this study was all-cause child mortality rate, it is important to underscore that not all of the under-5 deaths averted in PMI countries were due to malaria prevention and treatment. Nonetheless, we believe that the findings that coverage of various malaria prevention and treatment interventions—including ITNs, ACT, and IRS—increased in the years after PMI introduction provide a plausible mechanism for the decline in child mortality that was associated with PMI.

### Possible mechanisms

We examined existing evidence on the effectiveness of malaria interventions to assess whether the PMI-associated declines in child mortality were plausible. The widespread distribution of ITNs has previously been described as the most important malaria intervention in Africa [1, 34], accounting for 68% of the decrease in *P*. *falciparum* transmission rates between 2000 and 2015 [10]. A systematic review of efficacy trials from SSA found that use of ITNs reduced under-5 mortality by about a fifth [9]. An observational study in Kenya found that after mass distribution and promotion of ITNs, increased bednet use was associated with a 44% reduction in child mortality [32]. Consistent with other studies, we found that ITN coverage increased considerably in SSA since the turn of millennium, and that coverage was above average trends in PMI-recipient countries. Thus, our finding that PMI was associated with higher ITN coverage can partially explain the main finding regarding child mortality rates. We also found that PMI was associated with higher IRS coverage, particularly in the first few years of PMI program activities. Existing evidence shows that ITN and IRS coverage implemented together might have increased effectiveness, especially in high transmission areas with moderate ITN coverage [11, 21, 35]. Together, the rising coverage of ITNs and IRS may have offered an even greater protective effect on malaria burden and, ultimately, child mortality rates in PMI countries. Evidence also suggests that high coverage of ACTs may result in significant reductions in child mortality [33]. While the association between PMI and ACT coverage in our study was very modest, it is possible that higher access to malaria treatment might have made some, albeit small, contribution to the reduction in all-cause mortality. Our calculations showed the PMI-associated increases in ITN, IRS, and ACT coverage could account for a 12.5% reduction in all-cause child mortality. However, this result should be interpreted with caution given that we had to make several assumptions in our calculations and that we applied estimates from 2 specific settings to 32 different countries in SSA. Our calculations also did not account for interactive effects between ITNs, IRS, and ACTs or the fact that the increases in coverage detected in our study were considerably smaller in magnitude than those that were assumed or estimated in the other studies.

Other malaria interventions supported with PMI funds that were not assessed in this study, such as rapid diagnostic tests and IPTp, could have also contributed to reducing malaria burden and lowering all-cause child mortality rates. Protecting pregnant women from malaria in particular has been shown to lead to better birth outcomes and decreased risk of developing malaria, acute respiratory infection, and diarrhea during childhood, the leading causes of child deaths in SSA today [36, 37]. Finally, it is possible that introduction of PMI funds for malaria freed up domestic resources for other health interventions that further contributed to the reduction in all-cause child mortality. For instance, PMI invests in health systems strengthening and capacity building of laboratories and pharmaceutical chain systems. HIV programs have been found to have positive spillover effects to the broader health systems [38, 39], and it is possible that similar synergies exist between malaria-specific interventions and general health system functioning.

Internal and external evaluations of PMI have documented declines in under-5 mortality in PMI-recipient countries and concluded that the program was successful in making significant progress towards reducing child mortality [8, 18]. Our study provides additional evidence in support of this conclusion and extends the existing literature by using a quasi-experimental design. Despite PMI’s achievements, population coverage of key malaria interventions remained low throughout most of the African region [40]. As of 2015, most PMI countries were under target for key populations sleeping under ITNs and timely access to diagnostic tests and malaria medicines [8]. Furthermore, PMI has scaled down or even suspended IRS in some countries after worrying reports of resistance to insecticides have emerged [41, 42] and cost of other insecticides increased substantially [43]. The drop in IRS coverage in PMI recipient countries can be clearly seen in Figs 1 and 2. Bearing in mind that detecting population-level changes in ACT and IRS coverage can be difficult due to more targeted implementation, coverage of these interventions remained quite low throughout the study period, and increases in coverage after PMI implementation were also modest. As further support for malaria interventions is considered, health systems strengthening will be necessary to deliver more complicated interventions such as diagnostic tests and ACT, which require interacting with the healthcare system and a well-functioning drug supply chain system.

### Strengths and limitations

The reliance on 2 different measures of PMI activity and various robustness tests strengthened our confidence in the main study findings. It is reassuring that an association between PMI and the primary and secondary outcomes was detected using both a time-varying binary measure of receipt of PMI funding by a country (extracted from reports to Congress) and a continuous measure of per-capita measure of PMI funding obtained by countries in each year (obtained from IHME). Relying on disbursed dollars rather than committed dollars provided greater confidence that the exposure measure was correlated with interventions that reached populations in PMI countries. Confidence in the study findings was further strengthened by the robustness of results to adjustments for expenditures from all other funding sources captured in the IHME dataset. We were also reassured that exclusion of neonatal deaths, which could be attributed to better prenatal and delivery care rather than malaria interventions, amplified the magnitude of the association between PMI and child mortality. In addition, the data on coverage of malaria interventions were adjusted for geographic and temporal heterogeneity, which provides more robust malaria data [10, 40, 44]. Expanding the study sample to include additional comparison countries magnified the association between PMI and coverage of ITNs, ACTs, and IRS, suggesting that access to malaria interventions in the 9 additional countries (Botswana, Central African Republic, Equatorial Guinea, Eritrea, Guinea-Bissau, Mauritania, Somalia, South Sudan, and Sudan) was even more lacking than in the main study sample. The use of data spanning nearly 20 years enabled us to isolate the association between PMI and mortality from general shifts in child mortality trends in SSA and test the key assumptions of the difference-in-differences model. The finding that PMI and non-PMI countries had similar trends in child mortality rates prior to PMI introduction therefore strengthens the main finding regarding the association between PMI and child mortality rates. Lastly, PMI and comparison countries had similar governance characteristics when the program began, suggesting that it is unlikely that the program was selectively implemented in countries that were more favorable environments for malaria funding.

This study was subject to several limitations. First, difference-in-differences analysis relied on the assumption that there were no important unmeasured variables that differentially affected mortality rates in PMI and comparison countries during the study period. We used country fixed effects to control for all time-invariant differences between countries and year fixed effects to control for the underlying time trends in the region. Yet our study could still suffer from omission of important time-varying characteristics, which could bias our study results if the omitted variables affected PMI and comparison countries in different ways. For example, we were not able to account for national government spending on malaria interventions due to lack of reliable data. Countries have taken on more pronounced roles in funding their health systems and the potential contributions of domestic spending to the improved mortality rates should not be overlooked. Despite the increasing role of local governments globally, domestic malaria funding in SSA was fairly flat throughout the study period and, as of 2014, it still accounted for less than 10% of total malaria spending [1]. Nonetheless, PMI works in concert with recipient governments, and including national government contributions to fighting malaria would provide a fuller picture of which models of delivering malaria interventions have the highest value for money. Given the ongoing debate about how foreign aid affects recipient governments’ investments in the health sector [45–47], it is difficult to ascertain how governments might have adjusted their investment in malaria interventions after PMI was launched. This complicates the assessment of whether reallocation of resources was an important mechanism through which PMI was associated with all-cause mortality.

Second, the finding that Global Fund support was associated with only a modest reduction in all-cause child mortality, while consistent with one other study [48], should be interpreted with caution. The ability of our study to test hypotheses about the potential impact of this program was limited by the fact that all countries in our sample received Global Fund support by 2005. Thus, we did not have a comparison group of countries that did not receive Global Fund support, as we did for the evaluation of PMI. We suspect that the null finding can be largely explained by the lack of an appropriate comparison group that would have enabled us to test whether steady reductions in child mortality rates over time were due to Global Fund support or not. Our analysis of malaria interventions relied on the use of MAP data, which are modeled estimates. It is possible that the algorithm used to model the MAP data incorporated some inputs that were correlated with PMI. Finally, due to lack of suitable data, we were unable to evaluate whether the increase of IPTp (2 doses of sulfadoxine-pyrimethamine) coverage reported in PMI countries, from 14% at baseline to 38% in 2015 [8], are on par with or above the average trends in the rest of SSA. Future research should explore the potential impact of PMI on health behaviors in households with pregnant women.

### Implications

Our findings provide important new evidence of an accelerated decline in child mortality rates after the introduction of PMI. The investments that PMI funding enabled in key malaria interventions was associated with a large reduction in all-cause under-5 mortality, higher coverage of ITNs and IRS, and modest increases in ACTs. Further investment in the interventions supported with PMI funds may translate to additional lives saved, reduced household financial burdens associated with caring for ill household members and lost wages, and less strain on health systems associated with treating malaria cases. In other words, the health gains from PMI investment may have spillover effects beyond health, such as higher school attainment and labor productivity, which might in turn lead to greater economic development. Future research should explore whether investments made through programs such as PMI did in fact improve education and economic outcomes in SSA. Improved capacity of health systems, whether through health systems strengthening or recipient countries’ ability to shift their own resources to other health needs, might be a crucial component of PMI’s success.

## Supporting information

S1 Appendix Supplemental materials for “The US president’s malaria initiative and under-5 child mortality in sub-Saharan Africa”. (DOCX)

S1 IRB Notice (PDF)

S1 STROBE Checklist (DOCX)

## Abbreviations

ACT: artemisinin-based combination therapy
AIS: AIDS Indicator Surveys
DHS: Demographic and Health Surveys
IHME: Institute for Health Metrics and Evaluation
IPTp: intermittent preventive treatment in pregnancy
IRS: indoor residual spraying
ITN: insecticide-treated nets
MAP: Malaria Atlas Project
MIS: Malaria Indicator Surveys
PEPFAR: President’s Emergency Plan for AIDS Relief
PMI: President’s Malaria Initiative
RR: risk ratio
SSA: sub-Saharan Africa
