# Supplementary material for: The US President’s Malaria Initiative and under-5 child mortality in sub-Saharan Africa: A difference-in-differences analysis
Source: PLoS Med. 2017 Jun 13;14(6):e1002319. doi: 10.1371/journal.pmed.1002319 (PMC5469567; doi:10.1371/journal.pmed.1002319)
Supplement: S1 Appendix — (DOCX) [file pmed.1002319.s001.docx]

Supplemental materials for The US President’s Malaria Initiative and under-5 child mortality in sub-Saharan Africa: A difference-in-differences analysis

Table of contents

Description of sample and exposure 2

Data sources 2

*Child mortality data* 2

S1 Table A: Year of program implementation, year of DHS survey collection, and sample description 3

*Malaria Prevention and Treatment Technology Coverage* 3

*Program activity data* 4

Statistical Analysis 4

*Child mortality models* 4

*Malaria interventions models* 5

*Association between PMI and study outcomes over time* 6

*Parallel trends assumption test and sensitivity analysis* 7

S1 Table B: Descriptive characteristics of sample by PMI recipient and control countries 8

S1 Table C: Baseline under-5 mortality rate, PfPR transmission in 2-10 year olds, ITN coverage, ACT coverage, and IRS coverage in study countries. 9

Main Study Results displaying individual-level and household-level covariates 10

S1 Table D. Modified Poisson regression models of child mortality and implementation of large-scale healthcare interventions in sub-Saharan Africa from 1995 to 2014 10

S1 Table E. Modified Poisson regression models of child mortality and development assistance for health in sub-Saharan Africa from 1995 to 2012 11

Plausibility of study findings 12

Additional models of population coverage of malaria interventions and PMI program implementation 13

S1 Table F. Population coverage of insecticide treated nets (ITNs), artemisinin-based combination therapy (ACTs), and indoor residual spraying (IRS) in 19 PMI-recipient countries compared to 22 non-recipient countries in sub-Saharan Africa 13

S1 Table G. Association between child mortality/malaria intervention coverage and year of PMI program implementation 14

Robustness Checks 15

S1 Table H: Sensitivity analysis excluding neonatal deaths from the model (i.e. deaths before reaching one month of age) 15

S1 Table I: Child mortality trends after PMI program implementation stratified by urban/rural residence 16

S1 Table J: Sensitivity analysis excluding individual countries from the model 17

S1 Table K: Under-5 mortality trends using different model specifications: Modified Poisson, Cox, Logit, and Probit 18

S1 Table L: Parallel trends assumption using non-linear time trend 19

S1 Table M: Models of malaria interventions coverage using alternative data sources 20

S1 Fig A: Per capita disbursements for malaria from Global Fund and PMI over time 21

# Description of sample and exposure

The US President’s Malaria Initiative (PMI) began as a small program in 2006 with funding directed to three countries (Angola, Tanzania and Uganda) and an annual budget of $30 million. By 2011, PMI had expanded to 19 countries in SSA (Angola, Benin, Democratic Republic of Congo, Ethiopia, Ghana, Guinea, Kenya, Liberia, Madagascar, Malawi, Mali, Mozambique, Nigeria, Rwanda, Senegal, Tanzania, Uganda, Zambia, and Zimbabwe) and had an annual budget of approximately $600 million [1]. Our sample included all 19 PMI countries and 13 PMI comparison countries (Burkina Faso, Burundi, Cameroon, Chad, Congo, Cote d'Ivoire, Gabon, Namibia, Niger, Sierra Leone, Swaziland, The Gambia, and Togo). We excluded from the analysis malaria non-endemic countries (Lesotho), small island countries (Comoros, Sao Tome and Principe) and South Africa, since only one DHS from 1998 was available there. S1 Table A includes detailed information about when PMI started operating in each country and when other large funding sources, namely the Global Fund to Fight AIDS, Tuberculosis and Malaria (GFATM) and the US President’s Emergency Plan for AIDS Relief (PEPFAR), began operating in the study countries.

# Data sources

##

## ***Child mortality data***

Data on child mortality were extracted from every Demographic and Health Surveys (DHS) and Malaria Indicator Surveys (MIS) and AIDS Indicator Surveys (AIS) conducted between 1995 to 2014 that included child’s year and month of birth and age at death (in months). The DHS, MIS and AIS are standardized surveys collected by ICF International and funded by USAID that provide nationally-representative data on health and population in developing countries. Surveys were typically conducted every 5 years using standardized questionnaires with the goal of allowing for comparisons across countries and time. While some parts of the questionnaire have evolved over time, a core set of questions appeared in each data collection tool: household characteristics, woman characteristics, pregnancy/birth history. The child mortality data used in this study were obtained from birth history modules extracted from 77 DHS and 14 MIS and 5 AIS conducted between 1995 and 2014 [2].

DHS, MIS and AIS households were selected for participation in the survey using a 2-stage sampling design, where representative clusters were first selected from a national sampling frame and a random sample of households was then selected from within each cluster. Each survey thus yields a nationally-representative sample of households. In each sampled household, all women between 15 and 49 years old who were stable residents of the households were interviewed. Sampled women were asked detailed background characteristics and information on antenatal and postnatal care, place of delivery, who attended the delivery, birth weight, and the nature of complications during pregnancy for recent births. Women were also asked whether the child she gave birth to was still alive, and if not, when the child died.

We constructed a longitudinal cohort with repeated observations for each child who was <5 years of age between 1995 and 2014. We used information about whether each child was alive or dead, the year of child’s birth, and how old a child was when s/he died, when applicable, to create binary indicators of whether a child was deceased in each year. That is, we began with a set of women *W*_mjt_ sampled in country *j* and year *t.* We then restricted the sample to women who had ever given birth to a child and used information on child *i* date of birth to determine whether child *i* was between the ages of 0 and 5 years during study period (1995-2014), dropping all children older than 5 years old from the analysis. The three numbers *i, j, t* uniquely identified each child in each survey. For each eligible (<5 years) child *i* in our data we then generated a sequence of binary variables that indicated whether a child died in a given calendar year *t*. Our primary analytic dataset consisted of this sequence of binary variables, along with country characteristics for each year *t*, and all other characteristics that we knew about the child, mother and household from the index woman’s *W*_mjt_ survey responses (the child’s age and gender, mother’s age, mother’s education level, mother’s parity, household wealth, household was located in rural area, and whether female was the head of household). S1 Table A lists the number of child-year observations available per country, the number of unique children in our datasets and the number of deaths reported in each country. This analytic plan was similar to that used in other studies that have evaluated large-scale programs [3, 4].

## **S1 Table A: Year of program implementation, year of DHS survey collection, and sample description**

|  | Year Program Initiated | | |  | Sample Description | | | | |
| --- | --- | --- | --- | --- | --- | --- | --- | --- | --- |
|  | PMI | PEPFAR | Global Fund |  | Interview years | Number of child-year observations | Number of unique children | Number of deaths |  |
|  |  |  |  |  |  |  |  |  |  |
| PMI Countries | |  |  |  |  |  |  |  |  |
| Angola | 2006 | 2007 | 2005 |  | 2006-07, 2011 | 86,331 | 22,596 | 1,796 |  |
| Benin | 2008 |  | 2003 |  | 1996, 2001, 2006, 2011-12 | 370,331 | 102,199 | 8,077 |  |
| Congo, DRC | 2011 | 2007 | 2003 |  | 2007, 2013-14 | 295,437 | 77,322 | 8,295 |  |
| Ethiopia | 2008 | 2004 | 2003 |  | 2000, 2005, 2011 | 308,402 | 86,366 | 8,393 |  |
| Ghana | 2008 | 2007 | 2003 |  | 1998-99, 2003, 2008, 2014 | 164,164 | 44,705 | 3,169 |  |
| Guinea | 2011 |  | 2003 |  | 1999, 2005, 2012 | 184,426 | 52,235 | 5,997 |  |
| Kenya | 2008 | 2004 | 2003 |  | 1998, 2003, 2008-09, 2014 | 456,484 | 117,494 | 6,677 |  |
| Liberia | 2008 |  | 2004 |  | 2006-07, 2008-09, 2011, 2013 | 216,059 | 57,743 | 6,418 |  |
| Madagascar | 2008 |  | 2003 |  | 2003-04, 2008-09, 2011, 2013 | 245,162 | 66,524 | 3,410 |  |
| Malawi | 2007 | 2006 | 2003 |  | 2000, 2004-05, 2010, 2012, 2014 | 389,551 | 110,644 | 11,096 |  |
| Mali | 2008 |  | 2003 |  | 2001, 2006, 2012-13 | 337,077 | 95,958 | 12,035 |  |
| Mozambique | 2007 | 2004 | 2004 |  | 2003-04, 2011 | 201,349 | 55,698 | 5,900 |  |
| Nigeria | 2011 | 2004 | 2003 |  | 2003, 2008, 2010, 2013 | 815,253 | 216,519 | 27,219 |  |
| Rwanda | 2007 | 2004 | 2003 |  | 2000, 2005, 2010-11, 2013 | 234,697 | 66,684 | 6,717 |  |
| Senegal | 2007 |  | 2003 |  | 2005, 2008-09, 2010-11, 2012-13, 2014 | 568,322 | 149,602 | 12,714 |  |
| Tanzania | 2006 | 2004 | 2003 |  | 1999, 2004-05, 2007-8, 2009-10, 2011-12 | 291,498 | 81,687 | 5,643 |  |
| Uganda | 2006 | 2004 | 2003 |  | 2000-01, 2006, 2009-10, 2011 | 259,828 | 71,668 | 6,545 |  |
| Zambia | 2008 | 2004 | 2003 |  | 2001-02, 2007, 2013-14 | 285,167 | 75,298 | 6,621 |  |
| Zimbabwe | 2011 | 2006 | 2003 |  | 1999, 2005-06, 2010-11 | 128,460 | 35,882 | 1,829 |  |
| Comparison Countries | |  |  |  |  |  |  |  |  |
| Burkina Faso |  |  | 2003 |  | 1998-99, 2003, 2010, 2014 | 307,002 | 88,099 | 9,308 |  |
| Burundi |  | 2011 | 2003 |  | 2010-11, 2012-13 | 98,434 | 26,528 | 2,210 |  |
| Cameroon |  | 2011 | 2004 |  | 1998, 2004, 2011 | 218,262 | 61,086 | 5,522 |  |
| Chad |  |  | 2004 |  | 2004 | 47,646 | 13,931 | 1,771 |  |
| Congo |  |  | 2006 |  | 2005, 2011-12 | 147,884 | 39,342 | 3,001 |  |
| Côte d'Ivoire |  | 2004 | 2003 |  | 1998-99, 2011-12 | 103,514 | 27,894 | 2,902 |  |
| Gabon |  |  | 2004 |  | 2000-01, 2012 | 101,572 | 27,897 | 1,428 |  |
| Namibia |  | 2004 | 2004 |  | 2000, 2006-07, 2013 | 143,678 | 38,993 | 1,805 |  |
| Niger |  |  | 2004 |  | 1998, 2006, 2012 | 272,762 | 76,280 | 9,611 |  |
| Sierra Leone |  |  | 2003 |  | 2008, 2013 | 225,477 | 59,209 | 8,755 |  |
| Swaziland |  | 2007 | 2003 |  | 2006-07 | 28,425 | 7,838 | 572 |  |
| The Gambia |  |  | 2004 |  | 2013 | 96,713 | 24,370 | 1,397 |  |
| Togo |  |  | 2003 |  | 1998, 2013-14 | 122,704 | 34,660 | 2,648 |  |
| **Abbreviations:** PMI, President's Malaria Initiative; PEPFAR, President's Emergency Plan for AIDS Relief. | | | | | | | | | |

## ***Malaria Prevention and Treatment Technology Coverage***

We obtained data about population-level utilization of key malaria prevention and treatment interventions in sub-Saharan Africa from the Malaria Atlas Project (MAP), a non-profit organization at Oxford University, funded primarily by the Bill and Melinda Gates Foundation. The chief objective of MAP was to disseminate free, accurate and up-to date data on malaria and associated topics, organized on a geographical basis [5]. Coverage data from over one million households were combined with national malaria control program data on ITN, ACT and IRS provision to develop time-series models of coverage of these interventions within each country. Data sources included the Demographic Health Surveys (DHS), Malaria Indicator Surveys (MIS), Multiple Cluster Surveys (MICS), AIDS Indicator Surveys (AIS), Malaria and Anemia Prevalence Survey (EA & P), and the World Health Organization (WHO)[5].

The unit of analysis of MAP data was at the country-year level. ITN estimate represented the proportion of people who slept under an insecticide-treated bednet on any given night; ACTs estimate represented the proportion of fever cases in under-5 year olds that were treated with artemisinin-based combination therapy; IRS estimate represented the proportion of the population protected by indoor residual spraying of insecticides.

## ***Program activity data***

We extracted data about years when PMI began operations in each of the study countries from the PMI’s Tenth Annual Report to Congress (2016)[6]. Our study design accounted for the gradual enrollment of countries to receive PMI funding and for the fact that other large funding sources were present in the recipient countries during the study period. That is, we created an indicator for PMI program based on the actual year when funds were first disbursed, thus taking advantage of the variation in program rollout in the sample. We used the same strategy to create indicators for whether countries in the sample received PEPFAR and GFATM aid using publically-available government documents about program implementation[6-8].

The binary indicator of PMI program activity did not take into account that disbursement levels vary between countries and even within countries over time. In order to address this, we used data about the annual level of funds disbursed to each country to create a measure of program scope and intensity. These data were extracted from publically-available Development Assistance for Health 1990-2014 database developed by the Institute for Health Metrics and Evaluation (IHME) in Seattle WA[9]. IHME collected data from organizations that provided health-related funding in developing countries from 1990 through 2012. The dataset distinguished between the funding source (or country of origin), the channel through which funds were distributed (bilateral vs. multilateral vs. private foundations), health focus area (e.g. malaria, HIV) and where the funds were disbursed (or recipient country). This data structure enabled us to divide the development assistance for health to sub-Saharan Africa into six categories of interest.

The process to calculate per-capita disbursements was as follows: 1) we extracted the PMI disbursements in each country and year by summing all disbursements allocated to malaria interventions from the United States government through bilateral agreements; 2) we extracted funds from the Global Fund that were allocated specifically to malaria interventions; 3) we then grouped the remaining malaria aid from all other sources and channels into “other malaria aid” category; 4) we grouped Global Fund disbursements for HIV and tuberculosis; 5) we extracted PEPFAR disbursements by summing the funds allocated to HIV/AIDS interventions from the United States government through bilateral channels; and 6) we grouped all remaining development assistance for health into “other health aid” category. These six funding channels summed to 100% of health aid included in the IHME database as having provided funding to sub-Saharan Africa during the study period. In a final step we divided the disbursements in each of the six categories by the total country population in a given year, which we obtained from the World Development Indicators database [10]. Thus, the indicators that were included in the analysis represented per-capita development assistance for health in 2014 US dollars.

# Statistical Analysis

## ***Child mortality models***

First, we evaluated the association between PMI and all cause under-5 mortality by fitting modified Poisson regression models specified in Models (1-3):

(1) *Y_ijt_ = α_j_ + β* *POST_jt_ * PMI_j_ + γ_t_ +ε_ijt_*

(2) *Y_ijt_ = α_j_ + β* *POST_jt_ * PMI_j_ + µ POST_jt_ * GF_j_ + η POST_jt_ * PEPFAR_j_ + γ_t_ +ε_ijt_*

(3) *Y_ijt_ = α_j_ + β* *POST_jt_ * PMI_j_ + µ POST_jt_ * GF_j_ + η POST_jt_ * PEPFAR_j_ + λ Z_ijt_ + γ_t_ +ε_ijt_*

where *Y_ijt_* was the outcome variable set to 1 if a child *i* from country *j* in year *t* died and 0 otherwise. *POST_jt_*PMI_j_* variable was set to 1 if child *i* resided in country *j* that received PMI funds in year *t. POST_jt_*GF_j_* was set to 1 if child *i* resided in country *j* that received Global Fund aid in year *t. POST_jt_*PEPFAR _j_* was set to 1 if child *i* resided in country *j* that received PEPFAR funds in year *t. POST_jt_*GF_j_* and *POST_jt_* PEPFAR _j_* variables, which were added in Model (2), improved identification of PMI program by adjusting for the presence of other large funding sources to the region. Z*_ijt_ was* a vector of descriptive characteristics that was added in Model (3): child’s age (binary indicators for each year of child’s life), child’s gender (binary indicator for female), mother’s age (continuous measure of age), mother’s education (binary indicators for no education, primary education, and secondary of higher education), mother’s parity (continuous measure of the number of births given by the mother), urban/rural setting (binary indicator for rural), wealth index (binary indicators for wealth quintile of the household). Less than 5% of the study sample had missing data on household-level variable of wealth. We ran regression models using complete case analysis, i.e. observations with missing data were not used in the analysis. *α_j_ was* a full set of country dummies to control for baseline country characteristics, and γ*_t_* was a full set of year dummies to account for secular trends.

The coefficients of interest in Models (1-3) were *β’s*, which represented the difference-in-differences estimate of the PMI program, or the average annual change in risk of all-cause under-5 mortality in PMI countries. The exponentiated coefficient from Modified Poisson regression (i.e., Poisson regression with a robust error variance) provides a measure of relative risk[11, 12]. Standard errors were clustered at the country level to relax the assumption of independently and identically distributed error terms within countries [13].

Second, we evaluated the association between PMI program intensity and all-cause under-5 mortality by fitting modified Poisson regression models specified in Models (4-6):

(4) *Y_ijt_ = α_j_ + δ PC_PMI_aid_jt_ + γ_t_ +ε_ijt_*

(5) *Y_ijt_ = α_j_ + δ PC_PMI_aid_jt_ + φ PC_GF_MALARIA_aid_jt_ + μ PC_OTHER_MALARIA_aid_jt_ +*

*η PC_GF_HIV_TB_aid_jt_ + θ PC_PEPFAR_aid_jt_ + ρ PC_ALL_OTHERaid_jt_ + γ_t_ +ε_ijt_*

(6) *Y_ijt_ = α_j_ + δ PC_PMI_aid_jt_ + φ PC_GF_MALARIA_aid_jt_ + μ PC_OTHER_MALARIA_aid_jt_ +*

*η PC_GF_HIV_TB_aid_jt_ + θ PC_PEPFAR_aid_jt_ + ρ PC_ALL_OTHERaid_jt_ + λ Z_ijt_ + γ_t_ +ε_ijt_*

where *Y_ijt_* was the outcome variable set to 1 if a child *i* from country *j* in year *t* died and 0 otherwise. We began with Model 4 which only included the *PC_PMI_aid_jt_* variable, a continuous measure of the amount of aid disbursed through PMI in country *j* in year *t*. In Model (5) we added other continuous measures of per-capita spending to account for the presence and scale of other large-scale donors in the study countries. *PC_GF_MALARIA_aid_jtI_* variable measured the amount of aid from the Global Fund that was allocated towards malaria interventions in country *j* in year *t*. *PC_OTHER_MALARIA_aid_jt_* variable measured the amount of aid from all other sources allocated toward malaria interventions in country *j* in year *t*. *PC_GF_HIV_TB_aid_jt_* variable measured the amount of aid from the Global Fund that was allocated towards HIV and tuberculosis interventions in country *j* in year *t*. *PC_PEPFAR_aid_jt_* variable measured the amount of aid from the PEPFAR disbursed in country *j* in year *t*. *PC_ALL_OTHERaid_jt_* variable measured all other aid disbursed in country *j* in year *t*. All per-capita variables were continuous measures of aid in constant 2014 US dollars. Finally, in Model (6) we added Z*_ijt_* , a vector of descriptive characteristics: child’s age (binary indicators for each year of child’s life), child’s gender (binary indicator for female), mother’s age (continuous measure of age), mother’s education (binary indicators for no education, primary education, and secondary of higher education), mother’s parity (continuous measure of the number of births given by the mother), urban/rural setting (binary indicator for rural), wealth index (binary indicators for wealth quintile of the household). *α_j_ was* a full set of country dummies to control for baseline country characteristics and γ*_t_* was a full set of year dummies to account for secular trends.

The coefficients of interest in Model (4-6) were *δ’s*, which measured the average annual change in risk of all-cause under-5 mortality as a function of an additional per-capita PMI aid spent annually. The exponentiated coefficient from Modified Poisson regression provides a measure of relative risk. Robust standard errors were clustered at the country level to relax the assumption of independently and identically distributed error terms[13]. The other PC measures were included to help improve identification of PMI program activity by controlling for health aid disbursed through other channels in the same study countries and years, and individual-level characteristics were added to account for child’s, mother’s, and household characteristics that might have affected child’s risk of mortality.

***Malaria interventions models***

Third, we evaluated the association between PMI and population coverage of ITNs, ACTs, and IRS by fitting ordinary least squares (OLS) regression models specified in Model (7):

(7) *Y_jt_ = α_j_ +* ***λ*** *POST_jt_ * PMI_j_ + µ POST_jt_*GF_j_ + η POST_jt_*PEPFAR_j_ +π population_size_jt_ + γ_t_ +ε_jt_*

where *Y_jt_* were country-level continuous outcome variables: 1) percentage of population who slept under ITN on any given night; 2) percentage of cases of fever in under-5 year olds that were treated with ACT and 3) percentage of population protected by indoor residual spraying of insecticides. *POST_jt_ * PMI_j_*, *POST_jt_*GF_j_* and *POST_jt_*PEPFAR_j_* variables were specified as discussed above. *Population_size_jt_* was a time-varying continuous measure of population size in country *j* in year *t*. *α_j_* was a full set of country dummies to control for baseline country characteristics and γ*_t_* was a full set of year dummies to account for secular trends.

The coefficient of interest in Model (7) was *λ*, which represented the average annual percentage change in ITNs, ACTs and IRS coverage after implementation of PMI. Robust standard errors were calculated to correct for potential heteroskedasticity of the error term.

Fourth, we evaluated the association between PMI program intensity and population coverage of ITNs, ACTs and IRS by fitting ordinary least squares (OLS) regression model specified in Model (8):

(8) *Y_jt_ = μ PC_PMI_aid_jt_ + φ PC_GF_MALARIA_aid_jt_ + ν PC_OTHER_MALARIA_aid_jt_ +*

*η PC_GF_HIV_TB_aid_jt_ + θ PC_PEPFAR_aid_jt_ + ρ PC_ALL_OTHERaid_jt_ +π population_size_jt_ + γ_t_ +ε_ijt_*

where *Y_jt_* were country-level continuous outcome variables of population coverage of ITNs, ACTs, and IRS, as described above. All per-capita variables were continuous measures of aid in constant 2014 US dollars, as described above. *Population_size_jt_* was a time-varying continuous measure of population size in country *j* in year *t*. *α_j_* was a full set of country dummies to control for baseline country characteristics and γ*_t_* was a full set of year dummies to account for secular trends.

The coefficient of interest in Model (8) was *μ*, which represented the average annual percentage change in ITNs, ACTs and IRS coverage as a function of an additional per-capita PMI aid spent annually. Robust standard errors were calculated to correct for potential heteroskedasticity of the error term.

## ***Association between PMI and study outcomes over time***

We examined whether the association between PMI and all-cause under-5 mortality trends differed over time by fitting Model (9):

(9) *Y_ijt_ = α_j_ + χ PMIyear_jt_ + δ GFyear_jt_ + φ PEPFARyear_jt_ + λ Z_ijt_ + γ_t_ +ε_ijt_*

where *Y_ijt_* was the outcome variable set to 1 if a child *i* from country *j* in year *t* died and 0 otherwise. In these models, *POST*PMI* interaction term in Models (1-3) was substituted with a vector of binary variables indicating year of program implementation (e.g. *PMIyear1_jt_* set equal to 1 if PMI was in first year of implementation or 0 otherwise, *PMIyear2_jt_* set equal to 1 if PMI was in second year of program implementation or 0 otherwise, etc.). *GFyear_jt_* was a vector of binary indicators for year of Global Fund and *PEPFARyear_jt_* was a vector of binary indicators for year of PEPFAR program implementation. The coefficients of interest in Model (9) were a vector of *χ coefficients* which measured the annual relative risk change in child mortality as a function of PMI over time.

Lastly, we examined whether the association between PMI and population coverage of malaria intervention different over time by fitting Model (10):

(10) *Y_jt_ = α_j_ + σ PMIyear_jt_ + δ GFyear_jt_ + φ PEPFARyear_jt_ +π population_size_jt_ + γ_t_ +ε_ijt_*

where *Y_jt_* were country-level continuous outcome variables of population coverage of ITNs, ACTs, and IRS, as described above. *PMIyear_jt_* was a vector of binary variables indicating year of PMI program implementation (e.g. *PMIyear1_jt_* set equal to 1 if PMI was in first year of implementation or 0 otherwise, *PMIyear2_jt_* set equal to 1 if PMI was in second year of program implementation or 0 otherwise, etc.). The model also included a vector of binary indicators for year of Global Fund and PEPFAR program implementation. The coefficients of interest in Model (10) were a vector of *σ coefficients* which measured the annual percentage change in ITN, ACT and IRs coverage as a function of PMI over time.

## ***Parallel trends assumption test and sensitivity analysis***

The difference-in-differences design relies on the assumption that, in the absence of any intervention, countries receiving PMI funding would have identical *trends* in outcomes as non-recipient countries. We tested this “parallel trends” assumption by fitting the model shown in Model (11):

(11) *Y_ijt_ = α_j_ + β* *PMI_j_ + δ Year_t_ + γ PMI_j_ * Year_t_ + λ Z_ijt_ +ε_ijt_*

where *Y_ijt_* was the outcome variable set to 1 if a child *i* from country *j* in year *t* died and 0 otherwise. *PMI_j_* variable was set to 1 if child *i* resided in country *j* that was eventually selected to receive PMI funds. *Year_t_* was a linear time trend, in years. The coefficient of interest was *γ,* which shows whether the trends in countries that were eventually selected to receive PMI had different trends before the implementation of this intervention than the comparison countries. The coefficient *δ* showed the overall time trends in the SSA region before PMI was implemented and *β* compared baseline mortality between PMI and comparison countries. Z*_ijt_ was* a vector of descriptive characteristics: child’s age (binary indicators for each year of child’s life), child’s gender (binary indicator for female), mother’s age (continuous measure of age), mother’s education (binary indicators for no education, primary education, and secondary of higher education), mother’s parity (continuous measure of the number of births given by the mother), urban/rural setting (binary indicator for rural), wealth index (binary indicators for wealth quintile of the household). *α_j_ was* a full set of country dummies to control for differences between countries.

We performed several sensitivity analyses to verify our results. First, we excluded deaths that occurred in the first month of the child’s life to make sure that neonatal mortality was not driving our study results. Reductions in neonatal mortality can be attributed to better prenatal and delivery care rather than the malaria interventions that we wish to identify with our PMI models. Second, we tested whether results varied by area of residence. We hypothesized that the association between PMI and child mortality in rural areas would be stronger than in urban areas because malaria burden is much higher in rural areas. Third, we separately excluded each individual country from the analysis to ensure that no single country was driving the results. Fourth, we excluded Democratic Republic of Congo and Nigeria because PMI programs were implemented at sub-national scale in these two countries and under-5 mortality from malaria was especially high there [14]. We tested whether the results were robust to the type of model we ran by estimating logit, probit, and Cox models. We also confirmed that the parallel trends assumption held when we interacted a nonlinear time trend with PMI program indicators using data from pre-PMI years. Lastly, we confirmed that we were able to detect the association between PMI and malaria intervention coverage using alternative data sources

## **S1 Table B: Descriptive characteristics of sample by PMI recipient and control countries**

| Indicator | PMI  countries | Comparison  countries |
| --- | --- | --- |
| Number of child-year observations | 5,837,998 | 1,914,073 |
| Number of children in sample | 1,586,824 | 526,127 |
| Number of child deaths in sample | 148,551 | 50,930 |
| Female, % | 49.52 | 49.61 |
| Age < 1 year, % | 23.29 | 23.30 |
| Age < 2 years, % | 21.59 | 21.64 |
| Age < 3 years, % | 19.61 | 19.59 |
| Age < 4 years, % | 18.37 | 18.34 |
| Age < 5 years, % | 17.13 | 17.13 |
| Age at death, mean | 2.50 | 2.69 |
| Mother no education, % | 47.15 | 56.89 |
| Mother primary education, % | 36.89 | 23.54 |
| Mother secondary education, % | 14.10 | 18.23 |
| Mother higher education, % | 1.86 | 1.33 |
| Missing mother’s education, % | <1 | <1 |
| Mother's age, mean | 27.77 | 27.73 |
| Parity, mean | 3.50 | 3.45 |
| Wealth index, mean | 2.73 | 2.68 |
| Missing wealth index, mean % | 3.5 | 4.8 |
| Female household head, % | 20.35 | 19.81 |
| Rural, % | 73.30 | 68.67 |
| **Abbreviations:** %, Percent; PMI, President's Malaria Initiative  **Notes:** Sample based on Demographic and Health Surveys from 32 sub-Saharan countries from 1995-2014. PMI recipient countries: Angola, Benin, Congo DRC, Ethiopia, Ghana, Guinea, Kenya, Liberia, Madagascar, Malawi, Mali, Mozambique, Nigeria, Rwanda, Senegal, Tanzania, Uganda, Zambia. Comparison countries: Burkina Faso, Burundi, Cameroon, Chad, Congo, Cote d’Ivoire, Gabon, Namibia, Niger, Sierra Leone, Swaziland, The Gambia, and Togo. We report percentage of missing observations for the variables that have any missing data. | | |

## **S1 Table C: Baseline under-5 mortality rate, PfPR transmission in 2-10 year olds, ITN coverage, ACT coverage, and IRS coverage in study countries.**

|  | Under-5  mortality rate (per 1,000 live births)  in 2005 | Population weighted P*f*Pr_2-10_ in 2005 | ITN coverage  in 2005 | ACT coverage  in 2005 | IRS coverage  in 2005 |
| --- | --- | --- | --- | --- | --- |
| PMI countries | 121 | 30.0 | 10.3 | 1.5 | 2.9 |
| Angola | 204 | 27.9 | 4.9 | 0.7 | 3.6 |
| Benin | 126 | 41.1 | 7.9 | 0.3 | 0 |
| Congo, DRC | 138 | 61.7 | 6.0 | 0.4 | 0 |
| Ethiopia | 109 | 3.4 | 0 | 2.3 | 7.7 |
| Ghana | 87 | 41.7 | 10.9 | 1.6 | 0 |
| Guinea | 137 | 44.4 | 0.0 | 0.7 | 0 |
| Kenya | 86 | 10.9 | 19.2 | 0.4 | 1.7 |
| Liberia | 125 | 46.4 | 21.4 | 1.0 | 0 |
| Madagascar | 81 | 18.9 | 10.4 | 0.5 | 2.2 |
| Malawi | 116 | 26.1 | 26.4 | 2.7 | 0 |
| Mali | 172 | 49.6 | 0.0 | 4.1 | 0 |
| Mozambique | 134 | 42.3 | 4.9 | 0.9 | 0 |
| Nigeria | 158 | 43.1 | 0 | 0.5 | 0 |
| Rwanda | 111 | 4.7 | 14.3 | 1.8 | 0 |
| Senegal | 96 | 9.4 | 9.6 | 2.5 | 0 |
| Tanzania | 92 | 56.5 | 31.6 | 0.7 | 0 |
| Uganda | 107 | 20.5 | 16.2 | 3.6 | 0 |
| Zambia | 112 | 19.3 | 11.4 | 4.6 | 10.9 |
| Zimbabwe | 102 | 2.5 | 0.9 | 0.2 | 29.8 |
|  |  |  |  |  |  |
| Comparison countries | 128 | 30.2 | 3.5 | 1.6 | 2.3 |
| Burkina Faso | 158 | 55.4 | 0 | 0.1 | 0 |
| Burundi | 127 | 23.9 | 9.0 | 3.1 | 7.9 |
| Cameroon | 125 | 47.0 | 0 | 3.4 | 0 |
| Chad | 177 | 15.4 | 0 | 0.4 | 0 |
| Congo | 95 | 34.0 | 0 | 5.3 | 0 |
| Côte d'Ivoire | 129 | 67.9 | 0 | 0.6 | 0 |
| Gabon | 77 | 12.5 | 15.8 | 2.1 | 0 |
| Namibia | 98 | 8.8 | 0 | 1.2 | 21.9 |
| Niger | 72 | 26.7 | 0 | 1.9 | 0 |
| Sierra Leone | 173 | 49.7 | 0 | 1.2 | 0 |
| Swaziland | 204 | <1 | 0 | 0.2 | 0 |
| The Gambia | 130 | 6.7 | 20.6 | 0.1 | 0 |
| Togo | 105 | 44.5 | 0 | 1.1 | 0 |
| **Data sources:** World Development Indicators and Malaria Atlas Project.  **Abbreviations**: ITNs=estimated proportion of people who slept under an insecticide-treated bednet on any given night; ACTs=estimated proportion of cases of fever in under-5 year olds that were treated with Arteminisin Combination Therapy; IRS=estimated proportion of the population protected by indoor residual spraying of insecticides.  **Notes:** Under-5 mortality rate represents the probability per 1,000 that a newborn baby will die before reaching age five, if subject to age-specific mortality rates of the specified year. The numbers presented in row labeled “PMI countries” represent the mean values for PMI countries. The numbers presented on row labeled “Comparison countries” represent the mean values for comparison countries. | | | | | |

# Main Study Results displaying individual-level and household-level covariates

## **S1 Table D. Modified Poisson regression models of child mortality and implementation of large-scale healthcare interventions in sub-Saharan Africa from 1995 to 2014**

|  | Relative risk of  under-5 mortality  (1) | Relative risk of  under-5 mortality  (2) | Relative risk of  under-5 mortality  (3) |
| --- | --- | --- | --- |
|  | RR [95%CI] | RR [95%CI] | RR [95%CI] |
| *Implemented program* |  |  |  |
| Post PMI | 0.85** [0.74 - 0.96] | 0.84** [0.74 - 0.95] | 0.84** [0.74 - 0.96] |
| Post Global Fund |  | 0.95 [0.87 - 1.04] | 0.93 [0.85 - 1.02] |
| Post PEPFAR |  | 1.06 [0.96 - 1.17] | 1.05 [0.95 - 1.17] |
|  |  |  |  |
| *Child’s characteristics* |  |  |  |
| Female |  |  | 0.88*** [0.87 - 0.89] |
| Age (<1 year) |  |  | Ref. |
| Age < 2 years |  |  | 1.59*** [1.46 - 1.74] |
| Age < 3 years |  |  | 0.57*** [0.49 - 0.65] |
| Age < 4 years |  |  | 0.39*** [0.35 - 0.43] |
| Age < 5 years |  |  | 0.24*** [0.21 - 0.26] |
| *Mother’s characteristics* |  |  |  |
| No education |  |  | Ref. |
| Primary education |  |  | 0.90*** [0.86 - 0.94] |
| Secondary education |  |  | 0.79*** [0.75 - 0.82] |
| Higher education |  |  | 0.71*** [0.65 - 0.78] |
| Age |  |  | 0.94*** [0.93 - 0.94] |
| Parity |  |  | 1.18*** [1.17 - 1.19] |
| *Household characteristics* |  |  |  |
| Lowest wealth quintile |  |  | Ref. |
| Second wealth quintile |  |  | 0.99 [0.94 - 1.04] |
| Middle wealth quintile |  |  | 0.95* [0.91 - 1.00] |
| Fourth wealth quintile |  |  | 0.88*** [0.82 - 0.95] |
| Highest wealth quintile |  |  | 0.72*** [0.68 - 0.76] |
| Female household head |  |  | 1.02 [0.99 - 1.04] |
| Rural residence |  |  | 1.09*** [1.04 - 1.13] |
|  |  |  |  |
| No. observations | 7,752,071 | 7,752,071 | 7,404,578 |
| Country FE | Yes | Yes | Yes |
| Year FE | Yes | Yes | Yes |
| **Data Source:** Demographic Health Surveys.  **Abbreviations:** RR, risk ratio; 95% CI, 95% confidence interval; PMI, President's Malaria Initiative; PEPFAR, President's Emergency Plan for AIDS Relief.  **Notes:** Numbers 1, 2 and 3 refer to the Model number, specified in Statistical Analysis section. All models also included country fixed effects to control for baseline characteristics of countries and year fixed effects to control for secular trends. Standard errors were clustered at the country level. P-value notation: *** p<0.001, ** p<0.01, * p<0.05 | | | |

## **S1 Table E. Modified Poisson regression models of child mortality and development assistance for health in sub-Saharan Africa from 1995 to 2012**

|  | Annual risk of  under-5 mortality  (4) | Relative risk of  under-5 mortality  (5) | Relative risk of  under-5 mortality  (6) |
| --- | --- | --- | --- |
|  | RR [95%CI] | RR [95%CI] | RR [95%CI] |
|  |  |  |  |
| *Per-capita aid disbursements (US$)* |  |  |  |
| PMI (US bilateral aid for malaria) | 0.84*** [0.77 - 0.90] | 0.85*** [0.78 - 0.92] | 0.86*** [0.79 - 0.93] |
| Global Fund (malaria only) |  | 0.96* [0.93 - 1.00] | 0.96 [0.93 - 1.00] |
| Other aid for malaria |  | 1.04 [0.89 - 1.21] | 1.04 [0.87 - 1.24] |
| Global Fund (HIV/AIDS and TB) |  | 1.00 [0.96 - 1.03] | 1.00 [0.96 - 1.03] |
| PEPFAR (US bilateral aid for HIV/AIDS) |  | 1.01 [0.99 - 1.02] | 1.01 [0.99 - 1.02] |
| All other disbursements for health |  | 0.99 [0.98 - 1.01] | 1.00 [0.98 - 1.01] |
| *Child’s characteristics* |  |  |  |
| Female |  |  | 0.88*** [0.87 - 0.89] |
| Age (<1 year) |  |  | *Ref.* |
| Age < 2 years |  |  | 1.59*** [1.45 - 1.73] |
| Age < 3 years |  |  | 0.57*** [0.49 - 0.65] |
| Age < 4 years |  |  | 0.39*** [0.35 - 0.42] |
| Age < 5 years |  |  | 0.23*** [0.21 - 0.26] |
| *Mother’s characteristics* |  |  |  |
| No education |  |  | *Ref.* |
| Primary education |  |  | 0.90*** [0.87 - 0.94] |
| Secondary education |  |  | 0.79*** [0.76 - 0.82] |
| Higher education |  |  | 0.72*** [0.67 - 0.78] |
| Age |  |  | 0.94*** [0.93 - 0.94] |
| Parity |  |  | 1.18*** [1.17 - 1.19] |
| *Household characteristics* |  |  |  |
| Lowest wealth quintile |  |  | *Ref.* |
| Second wealth quintile |  |  | 0.98 [0.94 - 1.03] |
| Middle wealth quintile |  |  | 0.95 [0.91 - 1.00] |
| Fourth wealth quintile |  |  | 0.88*** [0.82 - 0.95] |
| Highest wealth quintile |  |  | 0.73*** [0.69 - 0.77] |
| Female household head |  |  | 1.02 [0.99 - 1.05] |
| Rural residence |  |  | 1.09*** [1.04 - 1.14] |
|  |  |  |  |
| No. observations | 7,140,735 | 7,140,735 | 6,829,406 |
| Country FE | Yes | Yes | Yes |
| Year FE | Yes | Yes | Yes |
| **Data Sources:** Demographic Health Surveys, Development Assistance for Health Database, and World Development Indicators.  **Abbreviations:** RR, risk ratio; 95% CI, 95% confidence interval; PMI, President's Malaria Initiative; PEPFAR, President's Emergency Plan for AIDS Relief.  **Notes:** Numbers 4, 5 and 6 refer to the Model number specified in Statistical Analysis section. All models also included country fixed effects to control for baseline characteristics of countries and year fixed effects to control for secular trends. Standard errors were clustered at the country level. P-value notation: *** p<0.001, ** p<0.01, * p<0.05 | | | |

# Plausibility of study findings

We used estimates from prior studies to calculate the plausible reductions in all-cause child mortality that would result from the PMI-associated increases in ITN, ACT, and IRS coverage that we reported in Table 5. These prior studies were conducted in two specific settings in sub-Saharan Africa. We used evidence from Kenya, where Fegan et al. (2007) estimated that increasing ITN coverage from 7% to 67% was associated with 44% reduction in all-cause child mortality [15]. We chose to rely on this study rather than results from efficacy trials [16] because the procedures of this study more closely resembled large-scale campaigns of ITN distribution that are supported with PMI funds than controlled experiments. Given the limited evidence about the impact of IRS on child mortality[17], we followed the example of Eisele et al. (2010) and assumed that IRS coverage had approximately equal protective effect to ITNs [18]. Finally, we used evidence from Zanzibar, where Bhattarai et al. (2007) found that reaching high coverage of ACTs (we used the conservative assumption that this implied full coverage) was associated 52% reduction in child mortality [19]. Applying these effect sizes of increased malaria intervention coverage on all-cause mortality to the PMI-associated increases in intervention coverage we obtained in our study (8.34 percentage point increase in ITN coverage, 6.63 percentage point increase in IRS coverage, and 2.98 percentage point increase in ACT coverage), we predicted the following reductions in all-cause child mortality: 6.12% from ITNs, 4.86% from IRS, and 1.55 from ACTs. The increased coverage of these three prevention and treatment modalities could therefore plausibly account for a 12.5% reduction in all-cause child mortality. These calculations should be interpreted with caution given the assumptions stated above, the reliance on estimates from two country settings to 32 different countries in SSA, and the fact that the increases in coverage detected in our study were considerably smaller in magnitude than those that were assumed or estimated in the other studies. Our calculations also did not account for interactive effects between ITNs, IRS and ACTs.

# Additional models of population coverage of malaria interventions and PMI program implementation

## **S1 Table F. Population coverage of insecticide treated nets (ITNs), artemisinin-based combination therapy (ACTs), and indoor residual spraying (IRS) in 19 PMI-recipient countries compared to 22 non-recipient countries in sub-Saharan Africa**

| Panel A (2000-2014) | Models of population coverage of malaria interventions and program implementation **in 19 PMI-recipient countries compared to 22 non-recipient countries** | | |
| --- | --- | --- | --- |
|  | ITN coverage | ACT coverage | IRS coverage |
|  |  |  |  |
|  | Coef. [95% CI] | Coef. [95% CI] | Coef. [95% CI] |
| *Implemented program* |  |  |  |
| Post PMI | 10.11** [3.49 - 16.73] | 5.64* [0.03 - 11.25] | 7.69** [2.42 - 12.96] |
| Post Global Fund | -4.74 [-12.25 - 2.77] | 1.34 [-3.37 - 6.05] | 1.37 [-2.79 - 5.54] |
| Post PEPFAR | -4.09 [-10.20 - 2.01] | 0.26 [-3.13 - 3.65] | -0.54 [-3.67 - 2.59] |
|  |  |  |  |
| No. observations (country-years) | 655 | 655 | 655 |
|  |  |  |  |
| Panel B (2000-2014) | Models of population coverage of malaria interventions and  per-capita disbursements for health **in 19 PMI-recipient countries compared to 22 non-recipient countries** | | |
|  | ITN coverage | ACT coverage | IRS coverage |
|  |  |  |  |
|  | Coef. [95% CI] | Coef. [95% CI] | Coef. [95% CI] |
| *Per capita aid disbursement (US$)* |  |  |  |
| US bilateral aid for malaria | 4.29* [0.54, 8.03] | 3.56 [-0.07, 7.19] | 1.98 [-1.32, 5.27] |
| Other aid for malaria | 9.69** [3.41, 15.97] | 1.70 [-4.40, 7.80] | 1.90 [-8.15, 11.95] |
| Global Fund (malaria only) | 1.51 [-0.02, 3.05] | 0.27 [-0.81, 1.35] | 0.11 [-1.33, 1.54] |
| Global Fund (HIV/AIDS and TB) | -0.22 [-0.77, 0.34] | -0.12 [-0.38, 0.15] | 0.61* [0.15, 1.08] |
| US bilateral aid for HIV/AIDS | -0.39* [-0.73, -0.04] | -0.03 [-0.26, 0.20] | -0.15 [-0.75, 0.45] |
| All other disbursements for health | -0.39 [-1.18, 0.39] | -0.15 [-0.74, 0.44] | 0.16 [-0.48, 0.79] |
|  |  |  |  |
| No. observations (country-years) | 527 | 527 | 527 |

**Data sources:** Malaria Atlas Project, Development Assistance for Health Database, and World Development Indicators.

**Abbreviations**: Coef.=coefficient; 95% CI=95% confidence interval; ITNs=estimated proportion of people who slept under an insecticide-treated bednet on any given night; ACTs=estimated proportion of cases of fever in under-5 year olds that were treated with Arteminisin Combination Therapy; IRS=estimated proportion of the population protected by indoor residual spraying of insecticides.

**Notes:** Coefficients can be interpreted as percent changes. Countries in the sample include all 19 PMI-recipient countries (Angola, Benin, Democratic Republic of Congo, Ethiopia, Ghana, Guinea, Kenya, Liberia, Madagascar, Malawi, Mali, Mozambique, Nigeria, Rwanda, Senegal, Tanzania, Uganda, Zambia, and Zimbabwe) and 22 PMI non-recipient countries (Botswana, Burkina Faso, Burundi, Cameroon, Chad, Central African Republic, Congo, Cote d'Ivoire, Equitorial Guinea, Eritrea, Gabon, The Gambia, Guinea-Bissau, Mauritania, Namibia, Niger, Sierra Leone, Somalia, South Sudan, Sudan, Swaziland, and Togo). Data from South Africa were also available but these were excluded because the country was an outlier (results with SA available from authors). All models also included country and year fixed effects and population size. Robust standard errors were used to calculate confidence intervals and p-values. P-value notation: *** p<0.001, ** p<0.01, * p<0.05.

## **S1 Table G. Association between child mortality/malaria intervention coverage and year of PMI program implementation**

| Annual risk of child mortality and year of PMI program implementation  (9) | |  | Population coverage of malaria interventions and year of PMI program implementation  (10) | | | |
| --- | --- | --- | --- | --- | --- | --- |
|  | Child mortality |  |  | ITNs | ACTs | IRS |
|  | RR [95%CI] |  |  | Coef [95%CI] | Coef [95%CI] | Coef [95%CI] |
| *Year of PMI program* |  |  | *Year of PMI program* |  |  |  |
| Year 1 | 0.93 [0.86 - 1.01] |  | Year 1 | 4.61 [-0.24 - 9.47] | 1.36 [-1.83 - 4.55] | 7.07* [1.31 - 12.83] |
| Year 2 | 0.82** [0.71 - 0.94] |  | Year 2 | 2.97 [-2.39 - 8.33] | 2.87 [-0.24 - 5.98] | 6.88** [1.97 - 11.79] |
| Year 3 | 0.77*** [0.66 - 0.89] |  | Year 3 | 6.17* [1.10 - 11.23] | 4.53 [-0.44 - 9.50] | 8.29** [2.84 - 13.74] |
| Year 4 | 0.73*** [0.61 - 0.88] |  | Year 4 | 7.85** [2.02 - 13.69] | 5.98* [1.44 - 10.52] | 9.97*** [4.43 - 15.50] |
| Year 5 | 0.65** [0.48 - 0.88] |  | Year 5 | 9.50** [2.82 - 16.19] | 5.26* [0.24 - 10.28] | 4.43 [-1.38 - 10.24] |
| Year 6 | 0.59* [0.37 - 0.94] |  | Year 6 | 12.51* [2.89 - 22.13] | 2.38 [-3.82 - 8.57] | 3.64 [-1.86 - 9.15] |
| Year 7 | 0.43 [0.15 - 1.25] |  | Year 7 | 16.52*** [7.45 - 25.60] | 5.68 [-0.65 - 12.00] | 5.81* [0.65 - 10.96] |
| Year 8 | 0.68 [0.17 - 2.71] |  | Year 8 | 17.37** [5.59 - 29.15] | 5.37 [-2.71 - 13.45] | 3.50 [-1.96 - 8.97] |
| Year 9 | Data not available |  | Year 9 | 6.04 [-17.94 - 30.02] | 16.16* [0.73 - 31.58] | -2.09 [-9.75 - 5.57] |
|  |  |  |  |  |  |  |
| No. observations (child-years) | 7,404,578 |  | No. observations (country-years) | 480 | 480 | 480 |
| GF year FE | Yes |  | GF year FE | Yes | Yes | Yes |
| PEPFAR year FE | Yes |  | PEPFAR year FE | Yes | Yes | Yes |
| Country FE | Yes |  | Country FE | Yes | Yes | Yes |
| Year FE | Yes |  | Year FE | Yes | Yes | Yes |
| Individual covariates | Yes |  | Population size | Yes | Yes | Yes |
| **Data Source:** Demographic Health Surveys, Development Assistance for Health Database.  **Abbreviations:** RR, risk ratio; 95% CI, 95% confidence interval; PMI, President's Malaria Initiative; PEPFAR, President's Emergency Plan for AIDS Relief.  **Notes:** All models also included country fixed effects to control for baseline characteristics of countries and year fixed effects to control for secular trends. Standard errors were clustered at the country level. P-value notation: *** p<0.001, ** p<0.01, * p<0.05 | | | | | | |

# Robustness Checks

## **S1 Table H: Sensitivity analysis excluding neonatal deaths from the model (i.e. deaths before reaching one month of age)**

| Models of child mortality and program implementation | |  | Models of child mortality and per-capita disbursements for health | |
| --- | --- | --- | --- | --- |
|  |  |  |  |  |
|  | RR [95%CI] |  |  | RR [95%CI] |
| *Implemented program* |  |  | *Per-capita aid disbursements (US$)* |  |
| Post PMI | 0.79*** [0.69 - 0.90] |  | PMI (US bilateral aid for malaria) | 0.83*** [0.75 - 0.92] |
| Post Global Fund | 0.95 [0.86 - 1.04] |  | Global Fund (malaria only) | 0.96 [0.93 - 1.00] |
| Post PEPFAR | 1.03 [0.92 - 1.16] |  | Other aid for malaria | 1.03 [0.84 - 1.26] |
|  |  |  | Global Fund (HIV/AIDS and TB) | 1.00 [0.96 - 1.03] |
|  |  |  | PEPFAR (US bilateral aid for HIV/AIDS) | 1.00 [0.99 - 1.02] |
|  |  |  | All other disbursements for health | 1.00 [0.98 - 1.01] |
| *Child’s characteristics* |  |  | *Child’s characteristics* |  |
| Female | 0.94*** [0.93 - 0.96] |  | Female | 0.94*** [0.92 - 0.96] |
| Age in years | 0.94*** [0.92 - 0.96] |  | Age in years | 0.94*** [0.92 - 0.96] |
| *Mother’s characteristics* |  |  | *Mother’s characteristics* |  |
| No education | Ref. |  | No education | Ref. |
| Primary education | 0.86*** [0.81 - 0.92] |  | Primary education | 0.87*** [0.82 - 0.92] |
| Secondary education | 0.71*** [0.67 - 0.75] |  | Secondary education | 0.71*** [0.67 - 0.76] |
| Higher education | 0.53*** [0.48 - 0.59] |  | Higher education | 0.54*** [0.49 - 0.59] |
| Age | 0.94*** [0.93 - 0.94] |  | Age | 0.94*** [0.93 - 0.94] |
| Parity | 1.19*** [1.17 - 1.20] |  | Parity | 1.19*** [1.17 - 1.20] |
| *Household characteristics* |  |  | *Household characteristics* |  |
| Lowest wealth quintile | Ref. |  | Lowest wealth quintile | Ref. |
| Second wealth quintile | 0.98 [0.93 - 1.04] |  | Second wealth quintile | 0.98 [0.92 - 1.04] |
| Middle wealth quintile | 0.94* [0.89 - 1.00] |  | Middle wealth quintile | 0.94 [0.89 - 1.00] |
| Fourth wealth quintile | 0.86** [0.78 - 0.94] |  | Fourth wealth quintile | 0.86** [0.78 - 0.95] |
| Highest wealth quintile | 0.64*** [0.59 - 0.70] |  | Highest wealth quintile | 0.65*** [0.59 - 0.71] |
| Female household head | 1.02 [0.99 - 1.05] |  | Female household head | 1.02 [0.99 - 1.05] |
| Rural residence | 1.12*** [1.07 - 1.17] |  | Rural residence | 1.12*** [1.07 - 1.17] |
|  |  |  |  |  |
| No. observations | 7,344,822 |  | No. observations | 6,920,625 |
| Country FE | Yes |  | Country FE | Yes |
| Year FE | Yes |  | Year FE | Yes |
| **Data Source:** Demographic Health Surveys, Development Assistance for Health Database.  **Abbreviations:** RR, risk ratio; 95% CI, 95% confidence interval; PMI, President's Malaria Initiative; PEPFAR, President's Emergency Plan for AIDS Relief.  **Notes:** All models also included country fixed effects to control for baseline characteristics of countries and year fixed effects to control for secular trends. Standard errors were clustered at the country level. P-value notation: *** p<0.001, ** p<0.01, * p<0.05 | | | | |

## **S1 Table I: Child mortality trends after PMI program implementation stratified by urban/rural residence**

| Association between PMI and under-5 mortality  by rural/urban area of residence | | |  | Association between aid disbursements and under-5 mortality  by rural/urban area of residence | | | |
| --- | --- | --- | --- | --- | --- | --- | --- |
|  | Rural | Urban |  |  | Rural | Urban |  |
|  | RR [95% CI] | RR [95% CI] |  |  | RR [95% CI] | RR [95% CI] |  |
| *Implemented program* |  |  |  | *Per-capita aid disbursements (US$)* |  |  |  |
| Post PMI | 0.83** [0.73 - 0.95] | 0.87* [0.76 - 1.00] |  | PMI (US bilateral aid for malaria) | 0.85*** [0.78 - 0.93] | 0.88*** [0.82 - 0.93] |  |
| Post Global Fund | 0.94 [0.84 - 1.06] | 0.93* [0.87 – 0.99] |  | Global Fund (malaria only) | 0.96* [0.92 - 0.99] | 0.98 [0.94 - 1.03] |  |
| Post PEPFAR | 1.03 [0.92 - 1.16] | 1.12 [0.99 - 1.27] |  | Other aid for malaria | 1.01 [0.83 - 1.21] | 1.11 [0.97 - 1.28] |  |
|  |  |  |  | Global Fund (HIV/AIDS and TB) | 0.99 [0.96 - 1.03] | 1.01 [0.99 - 1.03] |  |
|  |  |  |  | PEPFAR (US bilateral aid for HIV/AIDS) | 1.00 [0.99 - 1.02] | 1.01 [1.00 - 1.01] |  |
|  |  |  |  | All other disbursements for health | 0.99 [0.98 - 1.01] | 1.00 [0.98 - 1.01] |  |
|  |  |  |  |  |  |  |  |
| *Child’s characteristics* |  |  |  | *Child’s characteristics* |  |  |  |
| Female | 0.89*** [0.88 - 0.90] | 0.85*** [0.83 - 0.87] |  | Female | 0.89*** [0.88 - 0.90] | 0.84*** [0.82 - 0.86] |  |
| Age (<1 year) | *Ref.* | *Ref.* |  | Age (<1 year) | *Ref.* | *Ref.* |  |
| Age < 2 years | 1.68*** [1.54 - 1.83] | 1.33*** [1.21 - 1.47] |  | Age < 2 years | 1.67*** [1.53 - 1.83] | 1.33*** [1.20 - 1.47] |  |
| Age < 3 years | 0.62*** [0.53 - 0.71] | 0.41*** [0.37 - 0.46] |  | Age < 3 years | 0.62*** [0.53 - 0.71] | 0.41*** [0.36 - 0.46] |  |
| Age < 4 years | 0.43*** [0.39 - 0.47] | 0.29*** [0.26 - 0.32] |  | Age < 4 years | 0.42*** [0.38 - 0.46] | 0.28*** [0.25 - 0.32] |  |
| Age < 5 years | 0.25*** [0.23 - 0.27] | 0.19*** [0.17 - 0.22] |  | Age < 5 years | 0.25*** [0.23 - 0.27] | 0.18*** [0.16 - 0.20] |  |
| *Mother’s characteristics* |  |  |  | *Mother’s characteristics* |  |  |  |
| No education | *Ref.* | *Ref.* |  | No education | *Ref.* | *Ref.* |  |
| Primary education | 0.90*** [0.86 - 0.94] | 0.91*** [0.86 - 0.95] |  | Primary education | 0.91*** [0.86 - 0.95] | 0.91*** [0.87 - 0.96] |  |
| Secondary education | 0.79*** [0.75 - 0.84] | 0.77*** [0.73 - 0.82] |  | Secondary education | 0.79*** [0.75 - 0.84] | 0.78*** [0.73 - 0.82] |  |
| Higher education | 0.73*** [0.64 - 0.83] | 0.69*** [0.63 - 0.75] |  | Higher education | 0.74*** [0.67 - 0.81] | 0.70*** [0.64 - 0.76] |  |
| Age | 0.94*** [0.93 - 0.94] | 0.94*** [0.94 - 0.95] |  | Age | 0.94*** [0.93 - 0.94] | 0.94*** [0.94 - 0.95] |  |
| Parity | 1.18*** [1.17 - 1.19] | 1.18*** [1.16 - 1.20] |  | Parity | 1.18*** [1.17 - 1.19] | 1.18*** [1.16 - 1.20] |  |
| *Household characteristics* |  |  |  | *Household characteristics* |  |  |  |
| Lowest wealth quintile | *Ref.* | *Ref.* |  | Lowest wealth quintile | *Ref.* | *Ref.* |  |
| Second wealth quintile | 0.99 [0.93 - 1.04] | 1.00 [0.94 - 1.06] |  | Second wealth quintile | 0.98 [0.93 - 1.04] | 1.00 [0.94 - 1.06] |  |
| Middle wealth quintile | 0.96 [0.92 - 1.00] | 0.90** [0.85 - 0.97] |  | Middle wealth quintile | 0.96 [0.91 - 1.01] | 0.91** [0.85 - 0.97] |  |
| Fourth wealth quintile | 0.90** [0.83 - 0.96] | 0.82*** [0.75 - 0.90] |  | Fourth wealth quintile | 0.90** [0.84 - 0.97] | 0.82*** [0.75 - 0.90] |  |
| Highest wealth quintile | 0.74*** [0.69 - 0.79] | 0.69*** [0.64 - 0.76] |  | Highest wealth quintile | 0.75*** [0.70 - 0.79] | 0.70*** [0.64 - 0.76] |  |
| Female household head | 1.01 [0.98 - 1.04] | 1.05** [1.02 - 1.09] |  | Female household head | 1.01 [0.98 - 1.04] | 1.06** [1.02 - 1.09] |  |
| Rural residence | - | - |  | Rural residence | - | - |  |
|  |  |  |  |  |  |  |  |
| No. observations | 5,348,156 | 2,056,422 |  | No. observations | 4,939,048 | 1,890,358 |  |
| Country FE | Yes | Yes |  | Country FE | Yes | Yes |  |
| Year FE | Yes | Yes |  | Year FE | Yes | Yes |  |
| **Data Source:** Demographic Health Surveys, Development Assistance for Health Database.  **Abbreviations:** RR, risk ratio; 95% CI, 95% confidence interval; PMI, President's Malaria Initiative; PEPFAR, President's Emergency Plan for AIDS Relief.  **Notes:** All models also included country fixed effects to control for baseline characteristics of countries and year fixed effects to control for secular trends. Standard errors were clustered at the country level. P-value notation: *** p<0.001, ** p<0.01, * p<0.05 | | | | | | |  |

## **S1 Table J: Sensitivity analysis excluding individual countries from the model**

| Models excluding PMI countries | |  | Models excluding control countries | |
| --- | --- | --- | --- | --- |
|  | |  |  | |
| Excluded country | RR [95%CI] |  | Excluded country | RR [95%CI] |
| Angola | 0.83** [0.73 - 0.95] |  | Burkina Faso | 0.82** [0.72 - 0.93] |
| Benin | 0.83** [0.73 - 0.95] |  | Burundi | 0.82** [0.72 - 0.93] |
| Congo, DRC | 0.82** [0.72 - 0.94] |  | Cameroon | 0.84** [0.73 - 0.96] |
| Ethiopia | 0.84** [0.73 - 0.96] |  | Chad | 0.84** [0.74 - 0.96] |
| Ghana | 0.83** [0.73 - 0.95] |  | Congo | 0.83** [0.73 - 0.95] |
| Guinea | 0.84** [0.73 - 0.96] |  | Cote d'Ivoire | 0.84** [0.74 - 0.96] |
| Kenya | 0.83** [0.72 - 0.95] |  | Gabon | 0.84** [0.74 - 0.95] |
| Liberia | 0.85* [0.74 - 0.97] |  | Namibia | 0.85* [0.74 - 0.96] |
| Madagascar | 0.86* [0.76 - 0.97] |  | Niger | 0.83** [0.72 - 0.95] |
| Malawi | 0.84* [0.73 - 0.97] |  | Sierra Leone | 0.86* [0.76 - 0.98] |
| Mali | 0.84** [0.73 - 0.96] |  | Swaziland | 0.84** [0.74 - 0.96] |
| Mozambique | 0.83** [0.73 - 0.95] |  | The Gambia | 0.84** [0.73 - 0.95] |
| Nigeria | 0.84* [0.73 - 0.98] |  | Togo | 0.85* [0.75 - 0.97] |
| Rwanda | 0.85* [0.75 - 0.97] |  |  |  |
| Senegal | 0.84* [0.72 - 0.97] |  | Models excluding DRC and Nigeria | |
| Tanzania | 0.86* [0.76 - 0.98] |  | Excluded country | RR [95%CI] |
| Uganda | 0.83** [0.72 - 0.95] |  | Nigeria and DRC | 0.82* [0.70 - 0.97] |
| Zambia | 0.83** [0.73 - 0.95] |  |  |  |
| Zimbabwe | 0.84** [0.74 - 0.96] |  |  |  |
| **Data Source:** Demographic Health Surveys.  **Abbreviations:** RR, risk ratio; 95% CI, 95% confidence interval; PMI, President's Malaria Initiative.  **Notes:** The results listed here represent average annual change in risk of all-cause under-5 mortality in PMI countries when individual counties were excluded from the analysis. All models also included interactions for PEPFAR and GF programs, country fixed effects, year fixed effects, and individual-level covariates (i.e. Model 3). Standard errors were clustered at the country level. P-value notation: *** p<0.001, ** p<0.01, * p<0.05 | | | | |

## **S1 Table K: Under-5 mortality trends using different model specifications: Modified Poisson, Cox, Logit, and Probit**

|  | **Modified Poisson**  (glm, SE clustered at country) | **Cox**  (stcox, SE clustered at country) | **Logit**  (logit, SE clustered at country) | **Probit**  (probit, SE clustered at country) |
| --- | --- | --- | --- | --- |
|  | Coef. [95% CI] | Coef. [95% CI] | Coef. [95% CI] | Coef. [95% CI] |
| *Implemented program* |  |  |  |  |
| Post PMI | -0.18** [-0.31 - -0.05] | -0.18** [-0.31 - -0.05] | -0.18** [-0.31 - -0.04] | -0.07* [-0.12 - -0.01] |
| Post Global Fund | 0.05 [-0.06 - 0.16] | 0.05 [-0.06 - 0.16] | 0.06 [-0.06 - 0.16] | 0.02 [-0.02 - 0.07] |
| Post PEPFAR | -0.07 [-0.16 - 0.02] | -0.07 [-0.16 - 0.02] | -0.07 [-0.17 - 0.02] | -0.03 [-0.07 - 0.01] |
|  |  |  |  |  |
| *Child’s characteristics* |  |  |  |  |
| Female | -0.13*** [-0.14 - -0.11] | -0.13*** [-0.14 - -0.11] | -0.13*** [-0.15 - -0.12] | -0.06*** [-0.06 - -0.05] |
| Age (<1 year) | *Ref.* | *Ref.* | *Ref.* | *Ref.* |
| Age < 2 years | 0.47*** [0.38 - 0.55] | 0.53*** [0.42 - 0.64] | 0.49*** [0.40 - 0.58] | 0.22*** [0.17 - 0.26] |
| Age < 3 years | -0.57*** [-0.71 - -0.43] | -0.43*** [-0.59 - -0.27] | -0.59*** [-0.73 - -0.45] | -0.26*** [-0.31 - -0.20] |
| Age < 4 years | -0.94*** [-1.04 - -0.84] | -0.73*** [-0.85 - -0.62] | -0.96*** [-1.06 - -0.86] | -0.40*** [-0.44 - -0.36] |
| Age < 5 years | -1.45*** [-1.55 - -1.35] | -1.24*** [-1.36 - -1.12] | -1.47*** [-1.57 - -1.38] | -0.58*** [-0.62 - -0.55] |
| *Mother’s characteristics* |  |  |  |  |
| No education | *Ref.* | *Ref.* | *Ref.* | *Ref.* |
| Primary education | -0.11*** [-0.15 - -0.07] | -0.11*** [-0.15 - -0.07] | -0.11*** [-0.16 - -0.07] | -0.05*** [-0.07 - -0.03] |
| Secondary education | -0.24*** [-0.28 - -0.19] | -0.24*** [-0.28 - -0.19] | -0.25*** [-0.29 - -0.20] | -0.11*** [-0.13 - -0.08] |
| Higher education | -0.34*** [-0.43 - -0.25] | -0.34*** [-0.43 - -0.25] | -0.34*** [-0.44 - -0.25] | -0.14*** [-0.19 - -0.09] |
| Age | -0.06*** [-0.07 - -0.06] | -0.06*** [-0.07 - -0.06] | -0.07*** [-0.07 - -0.06] | -0.03*** [-0.03 - -0.03] |
| Parity | 0.16*** [0.15 - 0.18] | 0.16*** [0.15 - 0.18] | 0.17*** [0.16 - 0.18] | 0.07*** [0.07 - 0.08] |
| *Household characteristics* |  |  |  |  |
| Lowest wealth quintile | *Ref.* | *Ref.* | *Ref.* | *Ref.* |
| Second wealth quintile | -0.01 [-0.06 - 0.04] | -0.01 [-0.06 - 0.04] | -0.02 [-0.07 - 0.04] | -0.01 [-0.03 - 0.01] |
| Middle wealth quintile | -0.05* [-0.09 - -0.00] | -0.05* [-0.09 - -0.00] | -0.05* [-0.10 - -0.00] | -0.02* [-0.04 - -0.00] |
| Fourth wealth quintile | -0.13*** [-0.20 - -0.06] | -0.13*** [-0.20 - -0.06] | -0.13*** [-0.21 - -0.06] | -0.06*** [-0.09 - -0.03] |
| Highest wealth quintile | -0.33*** [-0.39 - -0.28] | -0.33*** [-0.39 - -0.28] | -0.35*** [-0.40 - -0.29] | -0.15*** [-0.17 - -0.12] |
| Female household head | 0.02 [-0.01 - 0.04] | 0.02 [-0.01 - 0.04] | 0.02 [-0.01 - 0.04] | 0.01 [-0.00 - 0.02] |
| Rural residence | 0.08*** [0.04 - 0.12] | 0.08*** [0.04 - 0.12] | 0.08*** [0.04 - 0.13] | 0.04*** [0.02 - 0.05] |
|  |  |  |  |  |
| No. observations | 7,467,239 | 7,467,239 | 7,467,239 | 7,467,239 |
| Country FE | Yes | Yes | Yes | Yes |
| Year FE | Yes | Yes | Yes | Yes |
| **Data Source:** Demographic Health Surveys, Development Assistance for Health Database.  **Abbreviations:** 95% CI, 95% confidence interval; PMI, President's Malaria Initiative; PEPFAR, President's Emergency Plan for AIDS Relief.  **Notes:** All models also included country fixed effects to control for baseline characteristics of countries and year fixed effects to control for secular trends. Standard errors were clustered at the country level. P-value notation: *** p<0.001, ** p<0.01, * p<0.05 | | | | |

## **S1 Table L: Parallel trends assumption using non-linear time trend**

|  | Annual risk of child mortality prior to PMI |
| --- | --- |
|  | RR [95% CI] |
| PMI-recipient country | 1.04 [0.96 – 1.13] |
| 1996 | 0.98 [0.92 - 1.04] |
| 1997 | 0.96 [0.86 - 1.06] |
| 1998 | 0.92 [0.81 - 1.04] |
| 1999 | 0.94 [0.82 - 1.08] |
| 2000 | 0.97 [0.87 - 1.08] |
| 2001 | 0.94 [0.82 - 1.07] |
| 2002 | 0.87* [0.78 - 0.98] |
| 2003 | 0.86* [0.74 - 0.99] |
| 2004 | 0.82** [0.73 - 0.93] |
| 2005 | 0.82* [0.70 - 0.97] |
| 2006 | 0.71** [0.57 - 0.89] |
| 2007 | 0.68*** [0.58 - 0.80] |
| 2008 | 0.63*** [0.53 - 0.75] |
| 2009 | 0.63*** [0.51 - 0.79] |
| 2010 | 0.57*** [0.45 - 0.73] |
| 2011 | 0.49*** [0.37 - 0.65] |
| 2012 | 0.37*** [0.23 - 0.58] |
| 2013 | 0.43** [0.26 - 0.72] |
| 2014 | 0.07** [0.01 - 0.40] |
| PMI-recipient country * 1996 | 0.99 [0.92 - 1.06] |
| PMI-recipient country * 1997 | 1.02 [0.91 - 1.13] |
| PMI-recipient country * 1998 | 1.05 [0.92 - 1.19] |
| PMI-recipient country * 1999 | 0.98 [0.85 - 1.13] |
| PMI-recipient country * 2000 | 0.94 [0.82 - 1.07] |
| PMI-recipient country * 2001 | 0.88 [0.75 - 1.03] |
| PMI-recipient country * 2002 | 0.90 [0.79 - 1.03] |
| PMI-recipient country * 2003 | 0.89 [0.76 - 1.05] |
| PMI-recipient country * 2004 | 0.87 [0.75 - 1.00] |
| PMI-recipient country * 2005 | 0.77* [0.63 - 0.95] |
| PMI-recipient country * 2006 | 0.87 [0.67 - 1.12] |
| PMI-recipient country * 2007 | 0.90 [0.71 - 1.13] |
| PMI-recipient country * 2008 | 0.94 [0.78 - 1.14] |
| PMI-recipient country * 2009 | 0.98 [0.76 - 1.26] |
| PMI-recipient country * 2010 | 1.06 [0.82 - 1.38] |
|  |  |
| No. observations (children-years) | 6,174,926 |
| **Data Source:** Demographic Health Surveys.  **Abbreviations:** RR, risk ratio; 95% CI, 95% confidence interval; PMI, President's Malaria Initiative.  **Notes:** PMI-recipient country variable indicates whether a country eventually received PMI funds. Year indicators represent non-linear time trends in under-5 mortality in the study sample. The coefficients of interest are the interactions of PMI country indicator and binary indicators of year, which measure whether mortality trends in countries that eventually received PMI differed from mortality time trend in comparison countries over time, adjusted for individual-level covariates. Model also included individual-level covariates: child’s age and gender, mother’s level of education, age and parity, rural/urban residence, household wealth and whether the head of household is female country fixed effects. Standard errors were clustered at the country level. Sample excludes observations from PMI-recipient countries after the program was implemented. P-value notation: *** p<0.001, ** p<0.01, * p<0.05 | |

## **S1 Table M: Models of malaria interventions coverage using alternative data sources**

| Panel A: World DataBank (country-level) | Sensitivity analysis 1 - World Bank Data | | |
| --- | --- | --- | --- |
|  | Percentage of children under age five who slept under an ITN to prevent malaria ^a^ | Percentage of children under age five who were ill with fever in the last two weeks and received any appropriate anti-malarial drugs ^a^ |  |
|  | Coef. [95% CI] | Coef. [95% CI] |  |
| *Implemented program* |  |  |  |
| Post PMI | 8.35* [0.40 - 16.30] | 1.16 [-7.64 - 9.97] |  |
| Post Global Fund | -5.26 [-14.94 - 4.42] | 4.55 [-6.02 - 15.12] |  |
| Post PEPFAR | -9.92 [-23.15 - 3.31] | 7.59 [-13.24 - 28.42] |  |
|  |  |  |  |
| No. observations (country-years) | 127 | 127 |  |
| Average in PMI countries before implementation | 10.6% | 39.9% |  |
|  | | | |
| Panel B: DHS, AIS, MIS data (household-level) | Sensitivity analysis 2 – DHS data | | |
|  | All children under-5 slept under net last night (household level) |  |  |
|  | Coef. [95% CI] |  |  |
| *Implemented program* |  |  |  |
| Post PMI | 13.1** [5.0 - 21.2] |  |  |
| Post Global Fund | -5.0 [-13.7 - 3.6] |  |  |
| Post PEPFAR | -7.0 [-15.7 - 1.6] |  |  |
|  |  |  |  |
| No. observations (household) | 410,261 |  |  |
| Average in PMI countries before implementation | 20.3% |  |  |
| **Data sources:** The World DataBank: UNICEF, State of the World's Children, Childinfo, and Demographic and Health Surveys.  **Abbreviations**: Coef.=coefficient; 95% CI=95% confidence interval.  **Notes**: Coefficients can be interpreted as percent changes. All models also included country and year fixed effects. Robust standard errors were used to calculate confidence intervals and p-values. P-value notation: *** p<0.001, ** p<0.01, * p<0.05. | | | |

## **S1 Fig A: Per capita disbursements for malaria from Global Fund and PMI over time**

**Supplement Materials References**

1. U.S. Agency for International Development. The President's Malaria Initiative: Eigth Annual Report to CongressApril 2014. Available from: <http://www.pmi.gov/docs/default-source/default-document-library/pmi-reports/pmireport_final.pdf?sfvrsn=16>.

2. ICF International. Demographic and Health Surveys (various). ICF International; 1995-2014. Available from: [http://dhsprogram.com/](http://dhsprogram.com).

3. Bendavid E, Holmes CB, Bhattacharya J, Miller G. HIV development assistance and adult mortality in Africa. JAMA. 2012;307(19):2060-7. Epub 2012/06/06. doi: 10.1001/jama.2012.2001. PubMed PMID: 22665105; PubMed Central PMCID: PMCPMC3434229.

4. Wagner Z, Barofsky J, Sood N. PEPFAR Funding Associated With An Increase In Employment Among Males in Ten Sub-Saharan African Countries. Health Affairs. 2015;34(6):946-53.

5. Bhatt S, Weiss D, Cameron E, Bisanzio D, Mappin B, Dalrymple U, et al. The effect of malaria control on Plasmodium falciparum in Africa between 2000 and 2015. Nature. 2015;526(7572):207-11.

6. U.S. Agency for International Development. The President’s Malaria Initiative’s (PMI’s) Tenth Annual Report to Congress 2016. Available from: <https://www.pmi.gov/docs/default-source/default-document-library/pmi-reports/pmi-tenth-annual-report-congress.pdf>.

7. The Global Fund to Fight AIDS TaM. Global Fund Disbursements 2015. Available from: <http://www.theglobalfund.org/en/data/datasets/>.

8. The President's Emergency Plan for AIDS Relief. PEPFAR dashboard [March 17 2016]. Available from: [https://data.pepfar.net/](https://data.pepfar.net).

9. Institute for Health Metrics and Evaluation (IHME). Development Assistance for Health Database 1990-2014. Seattle, United States.2015. Available from: <http://ghdx.healthdata.org/record/development-assistance-health-database-1990-2014>

10. The World Bank. World Development Indicators. 1995-2014 [February 2017]. Available from: [http://data.worldbank.org/](http://data.worldbank.org).

11. Zou G. A modified poisson regression approach to prospective studies with binary data. American journal of epidemiology. 2004;159(7):702-6.

12. Zou GY, Donner A. Extension of the modified Poisson regression model to prospective studies with correlated binary data. Statistical methods in medical research. 2013;22(6):661-70 %@ 0962-2802.

13. Bertrand M, Duflo E, Mullainathan S. How much should we trust differences-in-differences estimates? National Bureau of Economic Research, 2002.

14. Black RE, Cousens S, Johnson HL, Lawn JE, Rudan I, Bassani DG, et al. Global, regional, and national causes of child mortality in 2008: a systematic analysis. The lancet. 2010;375(9730):1969-87.

15. Fegan GW, Noor AM, Akhwale WS, Cousens S, Snow RW. Effect of expanded insecticide-treated bednet coverage on child survival in rural Kenya: a longitudinal study. The Lancet. 2007;370(9592):1035-9 %@ 0140-6736.

16. Lengeler C. Insecticide-treated bed nets and curtains for preventing malaria. Cochrane Database Syst Rev. 2004;2(2).

17. Pluess B, Tanser FC, Lengeler C, Sharp BL. Indoor residual spraying for preventing malaria. Cochrane Database Syst Rev. 2010;4(4).

18. Eisele TP, Larsen D, Steketee RW. Protective efficacy of interventions for preventing malaria mortality in children in Plasmodium falciparum endemic areas. Int J Epidemiol. 2010;39 Suppl 1:i88-101. Epub 2010/04/02. doi: 10.1093/ije/dyq026. PubMed PMID: 20348132; PubMed Central PMCID: PMCPMC2845865.

19. Bhattarai A, Ali AS, Kachur SP, Mårtensson A, Abbas AK, Khatib R, et al. Impact of artemisinin-based combination therapy and insecticide-treated nets on malaria burden in Zanzibar. PLoS Med. 2007;4(11):e309.
