# Supplementary material for: The US President’s Malaria Initiative and under-5 child mortality in sub-Saharan Africa: A difference-in-differences analysis
Source: PLoS Med. 2017 Jun 13;14(6):e1002319. doi: 10.1371/journal.pmed.1002319 (PMC5469567; doi:10.1371/journal.pmed.1002319)
Supplement: S1 IRB Notice — (PDF) [file pmed.1002319.s002.pdf]

From: **IRB** irb_no_reply@unc.edu
Subject: IRB Notice
Date: November 24, 2015 at 1:27 PM
To: aejaku@email.unc.edu
Cc: harsha@unc.edu, sylee@email.unc.edu

I

To: Aleksandra Jakubowski
Health Policy and Management Operations

From: Office of Human Research Ethics

Date: 11/24/2015
RE: Determination that Research or Research-Like Activity does not require IRB Approval
Study #: 15-3098

Study Title: Impact of US bilateral aid on population health in low-income countries

This submission was reviewed by the Office of Human Research Ethics, which has determined that this submission does not constitute human subjects research as defined under federal regulations [45 CFR 46.102 (d or f) and 21 CFR 56.102(c)(e)(l)] and does not require IRB approval.

Study Description:

Purpose: The goal of this research is to provide donor organizations and aid recipient countries with evidence on the effect of large-scale disease-specific aid programs on population health, health systems functioning and investments in human capital.

Participants: This study will use publically-available, de-identified data from the Demographic and Health Surveys.

Procedures (methods): We will use difference-in-differences study design to compare trends in all-cause child mortality, population-level utilization of healthcare services, and child school enrollment in countries that receive US bilateral aid compared to similar non-recipient countries.

Please be aware that approval may still be required from other relevant authorities or "gatekeepers" (e.g., school principals, facility directors, custodians of records), even though IRB approval is not required.

If your study protocol changes in such a way that this determination will no longer apply, you should contact the above IRB before making the changes.

CC:
Harsha Thirumurthy, Health Policy and Management Operations
Shoou-Yih Lee , Health Policy and Management OperationsIRB Informational Message - please do not use email REPLY to this address
